# Learning-Induced Plasticity in Medial Prefrontal Cortex Predicts Preference Malleability

**DOI:** 10.1016/j.neuron.2014.12.033

**Published:** 2015-01-21

**Authors:** Mona M. Garvert, Michael Moutoussis, Zeb Kurth-Nelson, Timothy E.J. Behrens, Raymond J. Dolan

**Affiliations:** 1 Wellcome Trust Centre for Neuroimaging, Institute of Neurology, University College London, London WC1N 3BG, UK; 2 Max Planck UCL Centre for Computational Psychiatry and Ageing Research, Russell Square House, 10-12 Russell Square, London WC1B 5EH, UK; 3 Oxford Centre for Functional MRI of the Brain, Nuffield Department of Clinical Neurosciences, University of Oxford, John Radcliffe Hospital, Oxford OX3 9D, UK

## Abstract

Learning induces plasticity in neuronal networks. As neuronal populations contribute to multiple representations, we reasoned plasticity in one representation might influence others. We used human fMRI repetition suppression to show that plasticity induced by learning another individual’s values impacts upon a value representation for oneself in medial prefrontal cortex (mPFC), a plasticity also evident behaviorally in a preference shift. We show this plasticity is driven by a striatal “prediction error,” signaling the discrepancy between the other’s choice and a subject’s own preferences. Thus, our data highlight that mPFC encodes agent-independent representations of subjective value, such that prediction errors simultaneously update multiple agents’ value representations. As the resulting change in representational similarity predicts interindividual differences in the malleability of subjective preferences, our findings shed mechanistic light on complex human processes such as the powerful influence of social interaction on beliefs and preferences.

## Highlights

• Learning the values of another causes plasticity in a mPFC value representation
• This plasticity predicts how much subjects’ own preferences change
• Plasticity is explained by a striatal surprise signal
• Value coding in mPFC occurs independently of the agent for whom a decision is made

## Teaser

Garvert et al. demonstrate that learning the preferences of another person increases the similarity between neural value representations for self and other. This plasticity in medial prefrontal cortex predicts how much one’s own preferences shift toward those of the other.

## Introduction

Information in the brain is encoded within distributed neuronal populations such that individual neurons typically support more than one representation or computation. Neurons in medial prefrontal cortex (mPFC), for example, perform self-referential as well as social value computations (Jenkins et al., 2008; Nicolle et al., 2012; Suzuki et al., 2012). Whereas it is traditionally suggested that computations for self and other are performed within separate populations of neurons (D’Argembeau et al., 2007; Denny et al., 2012), recent work suggests a functional organization within this region does not neatly conform to such a distinction by agent. Instead, value computations on behalf of any individual can be realized by the same circuitry (Nicolle et al., 2012), and the neural code depends only on the subjective value of an offer. In light of this, we conjectured that multiple value computations might be updated simultaneously if plasticity is introduced into this circuitry.

The contribution of overlapping neural circuitry to distinct computations has previously been demonstrated during delegated inter-temporal choice (Nicolle et al., 2012). In inter-temporal choice paradigms, subjects reveal their preferences for larger reward delivered later versus smaller reward that arrive sooner. Choice in this context is quantified by a “temporal discount rate” (Myerson and Green, 1995), believed to index forms of behavioral impulsivity (Evenden, 1999; Robbins et al., 2012) and an ability to imagine future outcomes (Ersner-Hershfield et al., 2009; Mitchell et al., 2011; Peters and Büchel, 2010). When subjects are asked to make such inter-temporal choices on behalf of another individual (“delegated inter-temporal choice”), they rapidly learn the confederate’s discount rate (Nicolle et al., 2012). This adaptability depends on the medial prefrontal cortex, where a neural circuitry used to compute a subject’s own values also computes those of a confederate, enabling rapid switches between the two computations (Nicolle et al., 2012).

We reasoned that if the same circuitry in the mPFC computes the value of a delayed offer irrespective of agents, plastic changes necessary to learn a new partner’s preferences might have consequences for a subject’s own value computations. The presence of such plasticity would also be expected to induce behavioral change in the subject’s own temporal discount rate, a parameter usually assumed to index a stable personality trait (Kirby, 2009; Ohmura et al., 2006). One can conjecture that such plasticity might underlie social conformity effects, where individuals adjust their beliefs or preferences to align more with those with whom they interact (Campbell-Meiklejohn et al., 2010; Edelson et al., 2011; Zaki et al., 2011).

At a neuronal level, a formal test of these predictions requires a fine-grained access to neural populations supporting distinct value computations, as well as a robust measure of learning-induced change in activity of these same populations. Despite its coarse spatial resolution, fMRI can reveal relationships between underlying cellular representations. In particular, fMRI adaptation paradigms can be finessed to measure plastic changes associated with the behavioral pairing of different items (Barron et al., 2013; Klein-Flügge et al., 2013). The principle of fMRI adaptation builds on the idea that the repeated engagement of the same neuronal population leads to a diminished response and attenuated BOLD signal, even though the underlying biophysical mechanism remains ambiguous (Grill-Spector et al., 2006; Kohn, 2007).

Here we used an fMRI adaptation paradigm to measure the relationship between neuronal value representations for self, a familiar other whose preferences had been learnt prior to scanning and a novel confederate as this latter agent’s preferences were learnt. We deployed a dynamic repetition suppression procedure to provide us with a probe of plastic neural changes associated with learning a new flexible computation. We hypothesized that plasticity associated with this new learning would impact upon the preference representation for self as a consequence of a neuronal representation that maps agent and offer onto an agent-independent measure of subjective value. In essence, this predicts that neuronal value representations between self and a novel other should become more similar with learning, in line with a behavioral shift in preference. An alternative hypothesis posits separate value computations for distinct agents. In such a case, a subject might use their own separate neural representations as a proxy for understanding another’s traits, and an independent neuronal value representation for this other would be constructed through learning-induced plasticity (Barron et al., 2013). This alternative scenario predicts that neural value representations for self and other should become less similar with learning. In terms of a mechanism driving such plasticity, we reasoned that the same prediction errors that drive learning about a new partner’s inter-temporal preferences would also induce shifts in the subject’s own discount rate toward that of the partner.

## Results

### Discount Rates Are Susceptible to Social Influence

**Figure 1:**
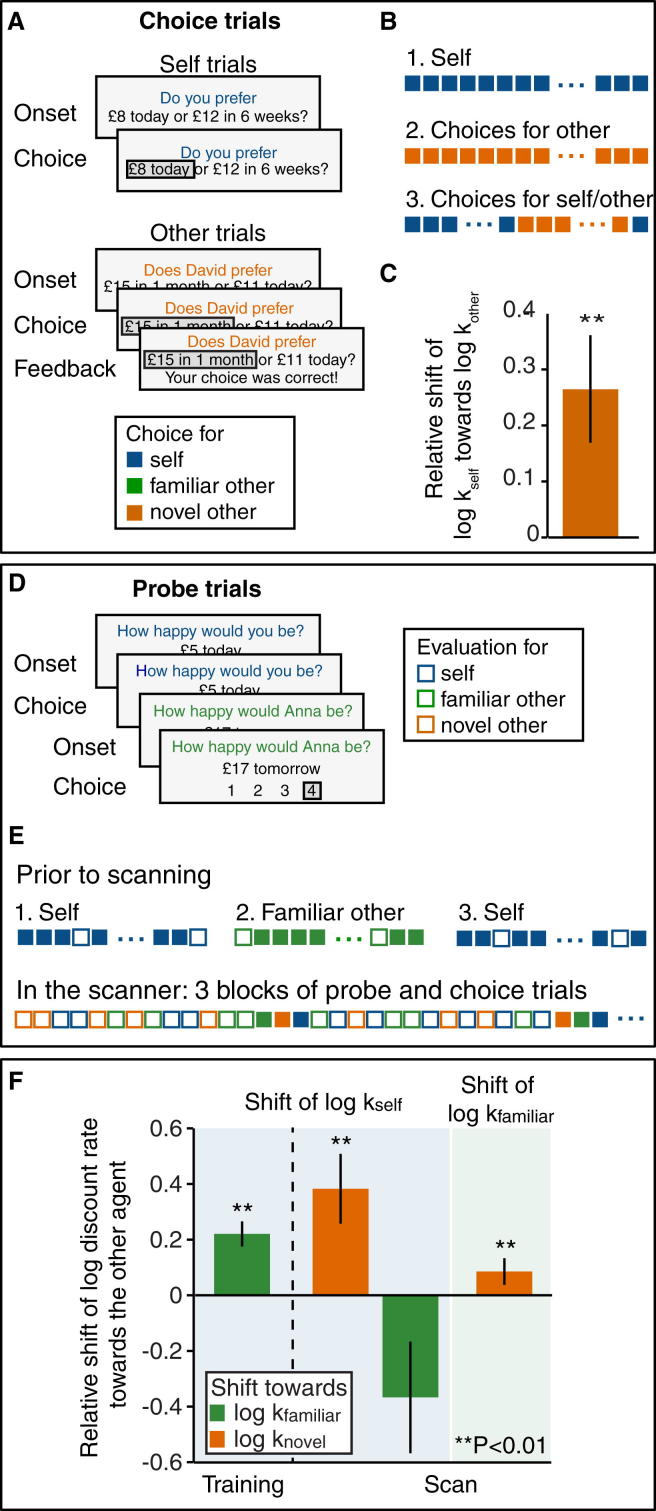
Experimental Design and Behavior (A) On choice trials, subjects chose between an immediately available, smaller, and a delayed, larger reward. On “self” trials, subjects considered the choice for themselves. On “other” trials, they made the choice on behalf of a partner, and feedback indicated whether their choice corresponded to the partner’s (simulated) choice. (B) Structure of the behavioral experiment. Block 1 consisted of self choice trials alone, block 2 consisted of other choice trials alone, and block 3 consisted of alternating short blocks of 10 choice trials per agent (self or other). (C) Shift of subjects’ own discount rate (block 3 − block 1) relative to the distance between the estimated discount rate of the partner (block 2) and the initial discount rate for self (block 1), $\text{shift}=\left(log\left({\text{k}}_{\text{self},\text{block}\;3}\right)-log\left({\text{k}}_{\text{self},\text{block}1}\right)\right)/\left(log\left({\text{k}}_{\text{other},\text{block}\;2}\right)-log\left({\text{k}}_{\text{self},\text{block}1}\right)\right)$. (D) The scanning version of the experiment also contained probe trials where subjects indicated on a four-item scale how happy an agent would be with the presented option. (E) Prior to scanning, subjects’ own discount rate was assessed before and after they were trained on the familiar other’s preferences. In the scanner, subjects chose and evaluated for themselves, for the familiar other and for a novel other. The experiment was divided into three experimental blocks with probe trials the predominant type in all blocks. (F) Relative shift of subjects’ own discount rate (blue background) and the discount rate of the familiar other (green background) toward the familiar other (green bars) and the novel other (orange bars) during training and scanning. Data are represented as mean ± SEM. See also Figure S1.

To examine whether learning about the preferences of another agent impacts on subjective inter-temporal preferences, we tested 27 subjects on a standard inter-temporal choice task both before, and after, performing the identical task on behalf of a partner (Figures 1A and 1B). As in the standard format, subjects deciding for themselves chose between an immediately available smaller reward and a delayed larger reward. The degree to which delay diminishes the value of a reward was then quantified by a discount rate, computed from each subject’s actual choices both before and after the experimental manipulation. The latter involved a context whereby subjects performed the very same task but now chose the option they inferred a confederate would prefer. After each trial they were given feedback about the choice the confederate had actually made, such that they could learn to simulate these choices in future trials.

Subjects learnt quickly, and accurately, to choose according to a novel partner’s preferences (Figures S1C and S1D). Subjects believed that the partner was a human participant playing the game in a neighboring room (Figures S1G and S1H). In actual fact, and in part motivated by a need for good experimental control, we delivered feedback of a simulated player with preferences very different from the subjects’ own (see Experimental Procedures).

Notably, we found that, after learning a partner’s preferences, subjects’ own discount rate shifted in the direction of the partner ($\left(\log\;k_{\mathrm{self},\mathrm{block}\;3}-\log\;k_{\mathrm{self},\mathrm{block}\;1}\right)/\left(\log\;k_{\mathrm{other},\mathrm{block}\;2}-\log\;k_{\mathrm{self},\mathrm{block}\;1}\right)$, t_21_ = 3.06, p = 0.006, Figure 1C). Their estimate of the novel other’s preferences remained stationary ($\left(\log\;k_{\mathrm{other},\mathrm{block}\;3}-\log\;k_{\mathrm{other},\mathrm{block}\;2}\right)/\left(\log\;k_{\mathrm{self},\mathrm{block}\;1}-\log\;k_{\mathrm{other},\mathrm{block}\;2}\right)$, t_21_ = 0.99, p = 0.33) and was not biased toward subjects’ own preferences (t_21_ = 0.49, p = 0.63). This effect is not easily understood as a social norm effect (Ruff et al., 2013), as we also observed discount rates shifted similarly when subjects were instructed they were deciding on behalf of a computer agent (t_22_ = 3.89, p < 0.001, Figure S1F).

One account of this shift in preference is that it arises out of a simulation of the other’s preferences. In order to test whether such simulation is crucial for this shift or whether the behavior can be explained by simple stimulus- or action-based reinforcement, we designed a category-learning control experiment (Ashby and Maddox, 2005). This consisted of the same stimuli and actions, but the necessity to simulate another’s discount rate was removed. Subjects were presented with a geometric depiction of a given choice on the screen (x axis: delay of the latter option; y axis: ratio of magnitudes M_LL_/M_SS_; Figure S1A, right) and instructed to choose according to the location of the dot with respect to an imaginary isoprobability line. Rather than using feedback to update a value simulation, subjects now updated their belief about the orientation of this line. In this scenario, subjects’ discount rates did not shift, indicating that subjects were not merely repeating previous choices they had made on behalf of the other (t_24_ = 0.61, p = 0.55; see Figure S1F). This latter finding emphasizes a necessity for preference simulation for another agent in order to modulate a discount rate.

### Subjective Value Changes Are Induced by Learning

The above account suggests that learning to compute the preferences of another agent induces plastic changes in the neural architecture responsible for personal valuation. This in turn predicts the neural population engaged during the computation of self valuation should change over the course of the experiment. This population should either become closer to that evoked during valuation for the partner if the representational structure of an offer depends solely on its subjective value irrespective of the individual. Alternatively, it should become less close if separate agent-specific representations exist and subjects construct an independent representation for the novel other as a consequence of learning. To test for such change in similarity between neural representations for self and others we interleaved trials from the delegated inter-temporal choice task with “probe” trials in the fMRI scanner. These probe trials enabled us to measure repetition suppression between individuals (Figures 1D and 1E). We reasoned that if self and partner valuation mechanisms overlapped more after learning than before, in line with an increase in behavioral similarity, then this predicts greater repetition suppression at the end of the experiment than at the beginning. If, however, subjects constructed a representation of the novel other from their representation of self, then this predicts the very opposite, namely repetition suppression at the beginning of the experiment, which disappears as subjects build a separate representation of the novel partner.

To be certain that any effects were driven by learning about the partner, as opposed to exercising a choice per se, we introduced a third player (a familiar partner) whose discount rate had been learnt prior to scanning. This controlled for non-specific time-dependent signal changes not associated with learning of new preferences. Thus, our experiment comprised three players: the subject (“self”), a partner whose preferences were learnt prior to scanning (“familiar other”), and a partner whose preferences were learnt during scanning (“novel other”). The familiar and novel others’ choices were simulated based on discount rates placed equally far apart on opposite, and counterbalanced, sides of the subject’s original discount rate. This meant that one partner had a smaller, and the other partner a larger, discount rate than the subject himself.

We scanned 27 subjects while they performed the two interleaved tasks. In choice trials, as in the behavioral experiment described above, subjects again made inter-temporal choices for themselves and for the two partners. In “probe trials,” subjects performed evaluations serially on behalf of different players, allowing us to measure repetition suppression between the value representations of different individuals (Figure 1E). After each choice trial for the novel or the familiar partner, but not after probe trials, subjects were given feedback about the choice the confederate had made.

In line with our behavioral results, subjects’ discount rates shifted toward the discount rate of the familiar partner during preference learning prior to scanning (t_23_ = 3.17, p = 0.004, Figure 1F). During scanning, both subjects’ own discount rate (t_23_ = 3.05, p = 0.006) and subjects’ estimated discount rate of the familiar partner (t_24_ = 2.87, p = 0.008) shifted toward the newly learnt discount rate of the novel partner, with a stronger relative shift evident for subjects’ own discount rate (t_22_ = 2.18, p = 0.04) but comparable absolute shifts (t_22_ = 0.72, p = 0.48). These preference shifts were therefore not simply associated with repeating the partner’s choices but instead are most parsimoniously explained as induced by learning a new individual’s preferences.

### Plasticity between Neural Representations of Self and Other

**Figure 2:**
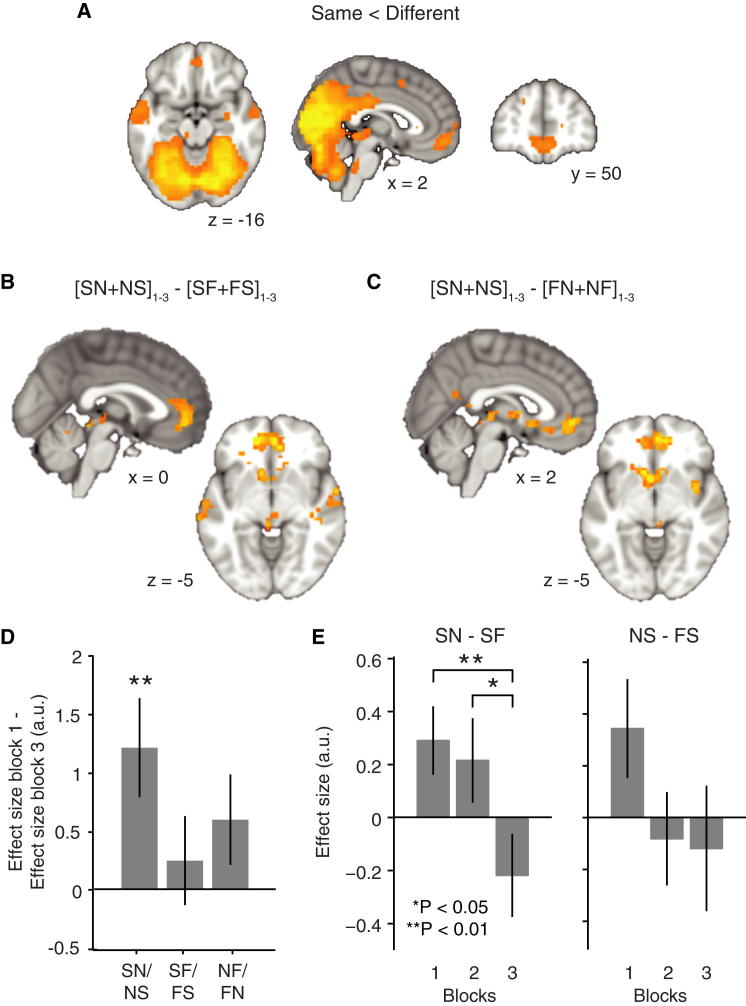
Learning-Induced Plasticity in mPFC (A) Repetition suppression as an index of representational similarity. Displayed are brain areas with significantly less activity for repeated compared to changing agents on subsequent trials. (B) Brain areas with a significantly greater increase in suppression from block 1 to block 3 between self and novel other compared to the increase in suppression between self and familiar other. This region overlaps with an area involved in self-referential processing and value coding (Figure S3). (C) Areas displaying an increase in suppression from block 1 to block 3 between self and novel other relative to changes in suppression between novel and familiar other. (D) Parameter estimates extracted by a jack-knife procedure from the mPFC ROI in Figure 2B, averaged across subjects. Visual areas do not show these selective suppression effects (Figure S2), and the neural suppression is not reflected in response times (Figure S4). (E) Same parameter estimates as in (D) but now separated into the distinct components. Data are represented as mean ± SEM. Contrast images in (A)–(C) are thresholded at p < 0.01 uncorrected for visualization. SN: novel-preceded-by-self; NS: self-preceded-by-novel; SF: familiar-preceded-by-self; FS: self-preceded-by-familiar; NF: familiar-preceded-by-novel; FN: novel-preceded-by-familiar. a.u.: arbitrary units.

To address whether a measured change in subjective preference is linked to plasticity in neural populations computing valuations for self, we focused our analysis on the probe trials. We first established that we could measure repetition suppression by comparing brain activity elicited by simulating values for an agent when preceded by the same agent compared to a situation where an agent was preceded by another agent. Different agents were indicated to the subject by different colors on screen (Figure 1D). Unsurprisingly, we observed fMRI adaptation in the visual cortex (p < 0.001, peak t_26_ = 16.93, [30, −61, −8], reported here and in subsequent fMRI analyses as familywise error (FWE) corrected on cluster level, Figure 2A) (Buckner et al., 1998; Wiggs and Martin, 1998), but also in a network that included mPFC (p = 0.02, peak t_26_ = 5.76, [3, 53, −11]) and left superior temporal sulcus (STS) (p < 0.001, peak t_26_ = 4.95, [−51, −13, −8]). The latter two regions are associated with mentalizing (Gallagher and Frith, 2003), valuation for self (Boorman et al., 2009; Hunt et al., 2012; Kable and Glimcher, 2007), and valuation for others (Jenkins et al., 2008; Nicolle et al., 2012). While this main effect of repetition suppression does not dissociate visual from agent-specific effects, it confirms that similarity in neural patterns evoked in a valuation network can be indexed by repetition suppression (Barron et al., 2013; Jenkins et al., 2008).

We reasoned that we could use this index of neural similarity to investigate whether the observed shift in subjective preferences was linked to plastic changes in the valuation network. If the neural code depends on the subjective values of a given offer alone, then repetition suppression should emerge between self and novel other over the course of the experiment, given that discount rates for self align with discount rates for the novel other. If, on the other hand, the mPFC encodes value differentially depending on agent, where learning another’s preferences involves the construction of an independent representation of this novel other from a representation of self, then repetition suppression should decrease over the course of the experiment. While a similar change in suppression might also be predicted between novel and familiar others, there should be no such change in suppression between self and familiar other if in fact we are indexing changes induced by new learning.

We designed a contrast that measured the change in repetition suppression between self and novel other from block 1 to block 3, controlled for by the change in repetition suppression between self and familiar other over the same blocks (see Experimental Procedures). The only brain region to survive whole-brain statistical correction was in mPFC (Figure 2B, p = 0.01, peak t_26_ = 3.82, [−12, 53, 1]), although sub-threshold clusters in the left and right STS were also present (p = 0.27, peak t_26_ = 3.77 and p = 0.48, peak t_26_ = 3.38, respectively). This region overlaps with an area involved in self-referential processing and in encoding value on probe trials (Figures S3B and S3C). There were no significant effects for the opposite interaction. This change cannot be due to visual effects, as we controlled for these both by inclusion of the familiar agent and separately by the comparison between early and late blocks in the experiment. Consequently, visual regions do not show these condition-specific changes in suppression (Figure S2). Neither can the effect be due to novelty or differences in processing speed, as no differences between main effects of novel and familiar others were seen in this region (Figure S3A) or in the response times (Figures S4A and S4B). Furthermore, an equivalent contrast measuring the change in suppression between self and novel other, but now controlling for the change in suppression between familiar and novel other, revealed a similar change in activity in an overlapping brain region (Figure 2C). Hence, in the mPFC learning the preferences of a novel agent specifically increased repetition suppression between representations of self and this novel partner.

To further investigate these mPFC suppression effects, we employed a jack-knife procedure across subjects to extract parameter estimates from the cluster of interest. Consistent with the whole-brain analysis, we found a significant change in novel-to-self/self-to-novel suppression (Figure 2D, t_26_ = 2.86, p = 0.008), but not in self-to-familiar/familiar-to-self suppression from block 1 to block 3 (t_26_ = 0.64, p = 0.52). The change in novel-to-familiar/familiar-to-novel suppression in the same region of interest (ROI) was in the same direction, but did not reach significance (t_26_ = 1.54, p = 0.14), and was smaller than the change in novel-to-self/self-to-novel suppression (t_26_ = 1.65, p = 0.05). Since overall activity in mPFC for self trials was greater than activity for other trials (Figure S3B), sensitivity to repetition suppression may differ depending on the order of the two agents. To explore potential differences, we decomposed the contrasts described above. Changes in repetition suppression between self and novel other were observed in both directions (Figure 2E) but were only significant when self trials were the priming and not the test trials (Figure 2E; ANOVA: left, F_2,78_ = 3.39, p = 0.04, right F_2,78_ = 1.55, p = 0.21).

### Plasticity in mPFC Predicts Discount Rate Shifts

**Figure 3:**
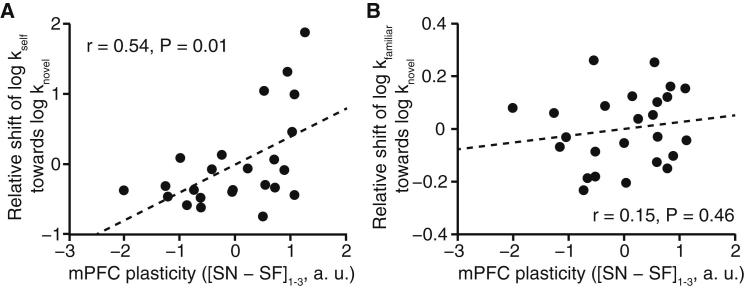
Relationship between [SN − SF]_1−3_ Plasticity and Shift in Discount Rate (A) Partial correlation between the change in suppression between self and novel relative to the change in suppression between self and familiar agents over blocks and the shift in subjects’ own discount rate toward the novel other. (B) Partial correlation between the change in suppression between self and novel relative to the change in suppression between self and familiar agents over blocks and the shift in subjects’ estimate of the familiar other’s discount rate toward the novel other. Parameter estimates in (A) and (B) were extracted from the mPFC ROI shown in Figure 2B. To account for the correlation between subjects’ own shift in discount rate and the shift in their estimate of the familiar other’s discount rate, we performed partial correlations (i.e., the familiar shift was removed from behavior and neural signal in [A] and the self shift was removed from behavior and neural signal in [B]). The relationship between [FN-SN]_1-3_ plasticity and the shift of the familiar other’s discount rate toward the novel other is analyzed in Figure S5. a.u.: arbitrary units.

If the observed behavioral change in preference is related to learning-induced plasticity in value computations, then the increase in representational similarity between self and novel other should predict a subject’s shift in preference. The increase in self-to-novel relative to self-to-familiar suppression over blocks did indeed predict the shift in subjects’ own discount rate toward the novel other (partial correlation, r = 0.54, p = 0.007, Figure 3A), but not the same shift in the subjects’ estimate of the familiar other’s discount rate (partial correlation, r = 0.15, p = 0.46, Figure 3B), although a direct comparison of these effects in a multiple regression analysis did not reach significance (t_23_ = 0.71, p = 0.24). The shift in subjects’ estimate of the familiar other’s preferences was instead loosely related to an increase in representational similarity between familiar and novel other (Figure S5). The fact that the behavioral estimate for a shift in discount rate was derived from choice trials, whereas the neural plasticity effect was extracted from probe trials, strongly suggests that learning a partner’s choice induces a stable plasticity in regions involved in value computation.

### Plasticity in mPFC Is Predicted by Surprise Coding in the Striatum

**Figure 4:**
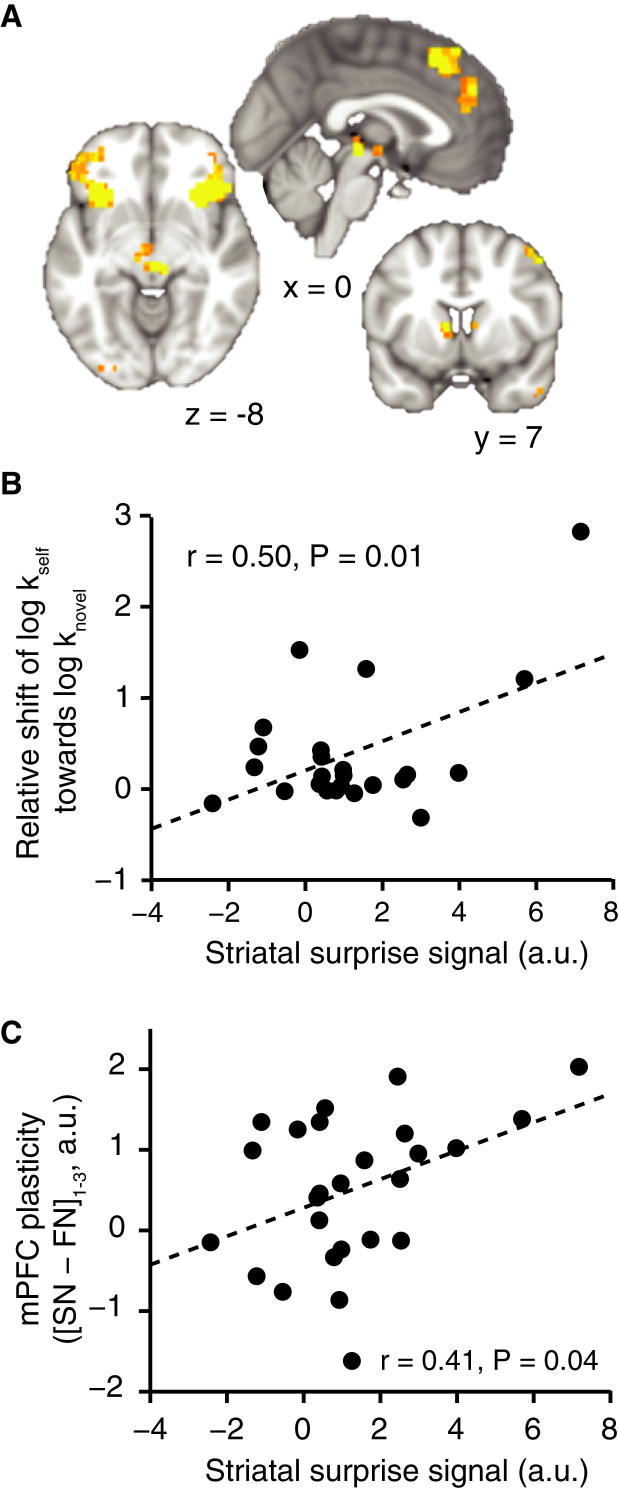
Surprise as a Mechanism Underlying mPFC Plasticity (A) Brain areas correlating with the surprise subjects experienced when observing the novel and the familiar partners’ choices. (B) Correlation between the striatal correlate of the surprise about the novel other’s choices, extracted from ROI in (A), and the shift of subjects’ discount rates toward the novel other. (C) Correlation between the striatal correlate of the surprise about the novel other’s choices and [SN − SF]_1−3_ plasticity in mPFC. (A) is thresholded at p < 0.01 uncorrected for visualization. a.u.: arbitrary units.

A plausible mechanism for inducing plastic change is surprise or prediction error, which in this context arises when the familiar or the novel partner’s choices diverge from the choice the subjects themselves would have made given the same choice context. Bayes-optimal estimates of this measure (see Experimental Procedures) were reflected in the posterior medial frontal cortex (pMFC) (Figure 4A, p = 0.04, peak t_26_ = 4.09, [−9, 29, 58]), a region previously associated with surprise coding in monkeys (Hayden et al., 2011), as well as in both insula and striatum (caudate nucleus), although these did not survive a stringent multiple comparisons correction (right insula: p = 0.16, peak t_26_ = 8.37, [30, 26, −8]; left insula: p = 0.19, peak t_26_ = 6.25, [−33, 26, −5]; left striatum (p = 0.84, peak t_26_ = 3.44, [3, −25, −8]). pMFC and striatum are strongly implicated in the expression of a prediction error type signal in reinforcement learning (Pessiglione et al., 2006; Voon et al., 2010), as well as in signaling a discrepancy between an individual’s behavior and the behavior of a group (Tomlin et al., 2013). An alternative measure of prediction error, where surprise was quantified as the discrepancy between the predicted choices of the partner and the partner’s actual choices, did not yield significant activity in any area of the brain. A more lenient cluster-defining threshold of p = 0.05 revealed much smaller clusters in a similar network as the first surprise measure that did not survive multiple comparisons correction (e.g. pMFC, p = 1.0, peak t_26_ = 2.72, [6, 35, 40]).

A striatal prediction error type signal is known to drive learning through an influence on cortico-striatal plasticity (Reynolds and Wickens, 2002). In line with this notion, the BOLD correlate of the surprise about the novel partner’s choices in the striatum predicted the behavioral shift in subjects’ own discount rate (Figure 4B, r = 0.50, p = 0.01) as well as the change in self-to-novel versus change in self-to-familiar neuronal suppression over blocks in mPFC (Figure 4C, r = 0.41, p = 0.04). No such relationship was evident for pMFC or insula activity and mPFC plasticity (r = 0.04, p = 0.84 and r = 0.14, p = 0.48, respectively).

**Figure 5:**
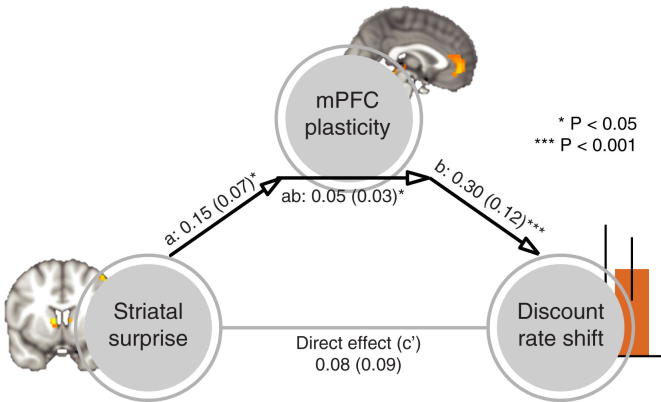
Mediation Path Diagram for Discount Rate Shift as Predicted from a Striatal Surprise Signal The striatal correlate of the surprise about the novel other’s choices predicted [SN − SF]_1−3_ plasticity in the medial prefrontal cortex (path a), and the mediator (mPFC plasticity) predicted the shift of subjects’ own discount rate toward the discount rate of the novel other (path b, controlled for the striatal surprise signal). Importantly, there was a significant mediation effect (path ab), indicating that mPFC plasticity formally mediates the relationship between striatal surprise and the shift in discount rate. The direct path between striatal surprise and shift in discount rate, controlled for both mediators, was not significant (path c'). The lines are labeled with path coefficients (SEs).

Finally, if prediction errors cause plasticity, and plasticity in turn causes the shift in subjects’ discount rate, then plasticity in mPFC should formally mediate the impact of the striatal surprise signal on the shift in discount rate. We used single-level mediation to test this hypothesis (Wager et al., 2008). The path model jointly tests three effects required if indeed mPFC plasticity provides the link between a surprise signal and the shift in discount rate: namely, the relationship between striatal surprise effects and mPFC plasticity (path a), the relationship between mPFC plasticity and shift in discount rate (path b), and a formal mediation effect (path ab) that indicates that each explains a part of the discount rate shift covariance while controlling for effects attributable to the other mediator. All three effects were significant in a mediation analysis (path a = 0.15, SE = 0.07, p = 0.04; path b = 0.30, SE = 0.12, p < 0.001; path ab = 0.05, SE = 0.03, p = 0.01, Figure 5), supporting the idea that prediction errors influence the discount rate by inducing mPFC plasticity, which in turn impacts upon choice behavior. Hence, subjects with the largest striatal surprise signal at outcome of choice trials exhibited both the largest changes in representational similarity on probe trials and the largest changes in preferences, suggesting a role for striatal prediction error signals in inducing cortical plasticity and associated behavioral change.

## Discussion

The brain’s representational architecture involves population codes wherein individual neurons contribute to a multitude of computations. We set out to investigate whether multiple neuronal representations can be updated simultaneously by learning-induced plasticity targeting one computation alone. The approach we developed exploited repetition suppression (Grill-Spector et al., 1999; Henson et al., 2000) to probe the similarity between distinct neural representations (Barron et al., 2013) by interleaving probe valuation trials with decision blocks that induced prediction errors and learning. While the biophysical mechanisms underlying fMRI repetition suppression remain ambiguous (Sobotka and Ringo, 1994), in a careful experimental design this approach allows inferences about population coding with respect to precise features of stimuli (Kourtzi and Kanwisher, 2001) or computations (Barron et al., 2013; Doeller et al., 2010).

We were interested in changes of value representational similarity over time. By asking subjects to evaluate presented options on behalf of themselves, a novel other whose preferences were acquired during on-line scanning and a familiar other whose preferences had previously been learnt, we could interrogate representational similarity in neuronal populations encoding valuation for these three agents. In line with previous reports that highlight a social influence on the valuation of objects (Campbell-Meiklejohn et al., 2010; Klucharev et al., 2009; Zaki et al., 2011), we found learning about the preferences of a novel agent had clear behavioral consequences evident in a shift in subjects’ own, as well as their estimation of a familiar other’s, discount rate. This behavioral effect coincided with an increase in neural representational similarity in the mPFC. This supports a view that value representations in the mPFC are not aligned to the frame of reference of an individual. Instead, the increase in neuronal overlap tied to a shift in behavioral preferences suggests that the mPFC encodes agent-independent representations of subjective value.

The presence of a learning-induced representational plasticity for value is likely to depend on generic learning mechanisms. The most influential computational account posits a role for a reward prediction error implemented via phasic activity of dopamine neurons (Schultz et al., 1997), a putative teaching signal for cortico-striatal learning (Calabresi et al., 2007; O’Doherty et al., 2004; Reynolds and Wickens, 2002). Prediction errors align with the dimension relevant for learning in a given situation. They manifest as a sensory prediction error when subjects learn to predict a sensory event (den Ouden et al., 2010), a probability prediction error when subjects learn about reward probability (Behrens et al., 2008), and a social expectancy prediction error when group preferences diverge from subjects’ own valuations (Campbell-Meiklejohn et al., 2010; Klucharev et al., 2009). In the current experiment, a prediction error (expressed in pMFC, insula, and striatum) corresponds to the surprise subjects experience when a partner’s choice is incongruent with their own preference. This accords with previous studies demonstrating an expression of a similar signal representing a discrepancy between one’s own and a group’s opinion (Berns et al., 2010; Campbell-Meiklejohn et al., 2010; Falk et al., 2010; Klucharev et al., 2009). Crucially, our results extend on these reports by showing this error coding is directly related to an expression of plasticity in mPFC, a region widely implicated in tracking preferences for stimuli (Lebreton et al., 2009) as well as inter-temporal preferences (Kable and Glimcher, 2007; Pine et al., 2009).

The mPFC region displaying the change in repetition suppression is a complex and heterogeneous area with strong connections to regions such as the amygdala, hippocampus, hypothalamus, and insula enabling access to sensory, visceral, and emotional information. It is considered ideally placed for self-referential processing (Kelley et al., 2002; Magno and Allan, 2007) and for attributing value to stimuli across many reward contexts (Bartra et al., 2013; Clithero and Rangel, 2014) and internally generated states (Bouret and Richmond, 2010). However, a mPFC value computation is also remarkably flexible, and can occur even if direct experience is not available (Barron et al., 2013) or if there is a requirement for an abstract model of task structure (Hampton et al., 2006). This flexibility is vital in social cognition, where a model of the preferences and intentions of another individual needs to be decoupled from the physical and perceptual reality of a subject’s own internal state (Mitchell, 2009; Nicolle et al., 2012). Traditionally, it has been suggested that such computations occur in distinct circuitries, where a ventral sector of the mPFC encoding subjective stimulus values (Boorman et al., 2009; O’Doherty, 2004) is complemented by a dorsal sector representing the mental states of others (Behrens et al., 2008, 2009; Frith and Frith, 2010; Yoshida et al., 2010). However, this notion is challenged by an observation that a dorsal-ventral axis can be better understood in terms of executed versus modeled choices (Nicolle et al., 2012). The latter observation supports the idea that the very same area encodes subjective value irrespective of the frame of reference, a notion strongly supported by our current observation that a behavioral shift toward the value of a novel agent is mirrored by an increase in neural overlap.

Irrespective of the exact nature of the observed plasticity, the underlying mechanism would seem to necessitate an overlap in neural populations encoding values for a novel other, self, and a familiar other. How exactly might the brain calculate discounting preferences with neural populations that are prone to the observed shifts in preference? Theoretical studies suggest an agent’s overall preferences might arise out of a summation over a distributed set of discounting units (Kurth-Nelson and Redish, 2009). This is consistent with recordings in rat orbitofrontal cortex demonstrating a distributed encoding of inter-temporal choice parameters across a neuronal population (Roesch et al., 2006). Similar gradients of discount factors have also been found in the human striatum (Tanaka et al., 2004) and mPFC (Wang et al., 2014). This suggests that some neuronal assemblies may represent a preference for fast discounting, favoring smaller-sooner returns, while others favor slow discounting. The discounting preference of each agent would be represented by population codes, implementing sets of weights over these discounting assemblies. The prediction errors a subject perceives when the novel other’s choices differ from what they would have chosen for themselves could in principle change the weights within this pool, resulting in altered populations codes.

The fact that a common brain region is recruited when computing preferences for self and other might suggest that people initially draw on self-representations to make inferences about another person and only construct a novel representation through learning. Such a mechanism has been observed when constructing a representation for a novel good from a simultaneous activation of familiar components (Barron et al., 2013). However, this theory makes opposite neural predictions, as it predicts repetition suppression at the beginning of the experiment as subjects draw on the same representation to choose for self and other. In this scenario a separate representation for a novel other is built over time and would predict disappearance of repetition suppression. Instead, we observe an increase in repetition suppression across time, an effect reminiscent of an increase in similarity between representations observed when subjects repeatedly evoke independent memories (Barron et al., 2013). Importantly, we can demonstrate this plasticity is not solely a neuronal phenomenon but also has profound behavioral consequences.

Our approach uses repetition suppression to provide insight into a similarity in neural representations. Comparable measures of representational content can be obtained by multivariate pattern analysis (Davis and Poldrack, 2013; Sapountzis et al., 2010); however, it is thought the two techniques show a difference in sensitivity to precise features of the neuronal code (Drucker and Aguirre, 2009). Without an explicit measure of MVPA in this study, we are therefore cautious in predicting a comparable increase in similarity between representations for self and a novel other in mPFC when using MVPA.

Note that subjects grow increasingly familiar with the novel other’s preferences as the task progresses, whereas familiarity remains constant for the familiar other in the sense that there is no new learning in relation to this other. Since psychological constructs such as familiarity, but also similarity and physical proximity, have previously been demonstrated to upregulate mPFC activity (Jenkins et al., 2008; Krienen et al., 2010; Mitchell et al., 2006; Tamir and Mitchell, 2011), this raises the question whether an increase in familiarity might drive the plasticity effect. Importantly, our data are not consistent with such an account. First, activity for familiar and novel other does not differ in mPFC, not even at the beginning of the experiment, suggesting that the mPFC in our task does not respond to familiarity per se. Second, a mediation analysis suggests that it is a striatal surprise signal, the very opposite of familiarity, that drives the plasticity effect, which in turn drives the behavioral shift.

Subjects’ own discount rate shifted toward the discount rate of their partner irrespective of whether their partner was human or a computer. This is in line with studies demonstrating that individuals use strategies akin to those used in real social contexts when interacting with a computer agent (Nass and Moon, 2000). Crucially, a control condition with the same stimuli and actions, but without the need to employ a discounting computation, did not evoke a change in subjects’ own preferences. This indicates that the behavioral effect is tied to subjects’ deployment of the very same discounting mechanism to learn on behalf of another agent, be it a human or non-human agent. Thus, it is a learning-induced plasticity in acquiring a novel value representation that impacted on subjects’ own subjective value computation. This also suggests that most subjects do not actively choose to change their preferences but instead do so as the consequence of an mPFC plasticity they are not consciously aware of. Such an implicit mechanism presumably contributes to involuntarily aligning goals with others and might play an important role in spreading values throughout a population (Boyd et al., 2011; Frith and Frith, 2010).

In conclusion, our data detail a neuronal mechanism by which personal traits are susceptible to social influence. Such plasticity might be one of the key features underlying learning, because it allows for an integration of past experience with novel information. More broadly, our findings pave the way for further studies of human social interactions at a more mechanistic level.

## Experimental Procedures

### Subjects

27 volunteers (mean age ± SD: 23.6 ± 3.7, 14 females) participated in the behavioral experiment, and 29 volunteers (mean age ± SD: 25.6 ± 5.6 years, 14 females) participated in the subsequent fMRI experiment. Two subjects were excluded from fMRI analyses, because they had previously participated in the behavioral experiment and because of technical difficulties during the scan. All subjects were neurologically and psychiatrically healthy. The study took place at the Wellcome Trust Centre for Neuroimaging in London, UK. The experimental procedure was approved by the University College London Hospitals Ethics Committee and written informed consent was obtained from all subjects.

### Task Behavioral Study

For a detailed description of the task and our analyses, see the Supplemental Information. In brief, subjects made a series of choices between a smaller amount paid on the same day and a larger amount paid later (Figure 1A). The experiment was divided into three blocks (Figure 1B). In the first block, consisting of 100 trials, subjects made decisions for themselves. In block 2, they made decisions on behalf of their partner. They were also provided with trial-by-trial feedback on whether their choice for the partner was correct. Block 2 ended when subjects made 85% correct responses for their partner in a sliding window of 20 trials or after a maximum of 60 trials. In block 3, smaller blocks of ten trials of choosing for self alternated with blocks of ten trials of choosing for the partner. Block 3 ended after a total of 200 trials. Choices were optimized to give us a precise estimate of subjects’ discount rates.

### Estimation of Discount Rates

We estimated subjects’ discount rates by fitting a hyperbolic model to their choices (Rachlin et al., 1991) separately for each experimental block. Subjects’ shift in discount rates was defined as the change in discount rate from block 1 to block 3 (log k_self,block3_ − log k_self,block1_) relative to the distance between their estimate of the partner’s discount rate from their own discount rate (log k_other,block2_ − log k_self,block1_):

$$\left(1\right)\;shift=\frac{log\;k_{self,block\;3}-log\;k_{self,block1}}{log\;k_{other,block\;2}-log\;k_{self,block1}}.$$

A positive shift represents a movement toward, and a negative shift a movement away from, the partner’s discount rate. Outliers (outside the range mean ± 3⋅SD), as well as subjects who estimated their partner’s discount rate to be less than 0.3 units away from their own discount rate, were excluded from population analyses because of inflated shift estimates (see Figure S1E).

### Simulation of the Other’s Choices

To generate feedback for the confederate’s choices, we simulated a partner with a discount rate that differed from the subject’s own baseline discount rate by 1 (i.e., log k_other_ = log k_self,block1_ ± 1). Choices were correct if they corresponded to the decision that would be preferred by a hyperbolic discounter with this discount rate. Importantly, the simulated partner’s choices were noisy, as the other’s subjective value was translated to a choice probability with a softmax function (temperature parameter β = 1).

### Task fMRI Study

The fMRI experiment consisted of two trial types: choice trials, as described for the behavioral experiment above, and probe trials, in which subjects evaluated a single option on a scale from 1 to 4 (Figure 1D). Subjects learned the preferences of a second partner (“familiar other”) before the scan (Figure 1E, top).

In contrast to the behavioral experiment and the pretraining, subjects learned about the novel other’s discount rate while we assessed their own discount rate. To make sure that we captured a potential shift in discount rate in this scenario, we excluded the first third of all choice trials subjects performed in the scanner when estimating k_self,scan_, k_novel,scan_, and k_familiar,scan_. The relative shift effects reported in Figure 1F were then calculated as follows:

$$\left(4\right)\;shift_{self\to fam,training}=\;\frac{\text{log}\;k_{self,training\_block\;3}-\log\;k_{self,training\_block1}}{\log\;k_{familiar,training\_block2}-\log\;k_{self,training\_block1}}$$

$$\left(5\right)\;shift_{self\to fam,scan}=\frac{\text{log}\;k_{self,scan}-\log\;k_{self,training\_block3}}{\log\;k_{familiar,scan}-\log\;k_{self,training\_block3}}$$

$$\left(6\right)\;shift_{self\to novel,scan}=\frac{\text{log}\;k_{self,scan}-\log\;k_{self,training\_block3}}{\log\;k_{novel,scan}-\log\;k_{self,training\_block3}}$$

$$\left(5\right)\;shift_{fam\to novel,scan}=\;\frac{\text{log}\;k_{familiar,scan}-\log\;k_{familiar,training\_block2}}{\log\;k_{novel,scan}-\log\;k_{familiar,training\_block2}}.$$

For the estimation of absolute shifts, the denominator z was set to sign(z). Outliers (outside the range mean ± 3^∗^SD) as well as subjects for whom the denominator was smaller than 0.3 (two subjects for shift_self→fam,scan_, three subjects for shift_self→novel,scan_, and two subjects for shift_fam→novel,scan_) were excluded from the analyses.

### Surprise Measure

We estimated subjects’ own discount rates on a trial-by-trial basis (see Supplemental Information) and used this measure to compute differences in subjective value for the choices subjects observed their partner make (V_chosen_by_partner_ − V_unchosen_by_partner_). This difference in subjective value was transformed to a probability using a softmax function applied to a trial-to-trial estimation of subject’s inverse temperature parameter β. This measure gave us an estimate of how likely the subject would have been to make the same choice himself. We subtracted this likelihood from 1 to translate this to a surprise measure.

### Scan Procedure, fMRI Data Acquisition, and Preprocessing

We used standard procedures for acquiring fMRI data where these were designed to minimize susceptibility related artifacts in the ventral prefrontal cortex (Weiskopf et al., 2006). We used SPM8 for image preprocessing and data analysis (Wellcome Trust Centre for Neuroimaging, London). We corrected for signal bias, co-registered functional scans to the first volume in the sequence, and corrected for distortions using the fieldmap. Data were spatially normalized to a standard EPI template and smoothed using an 8 mm full-width at half maximum Gaussian kernel.

### fMRI Data Analysis

Data were analyzed with an event-related general linear model (GLM). Probe trials were sorted into nine different conditions (self preceded by self [SS], novel preceded by self [SN], familiar preceded by self [SF], self preceded by novel [NS], novel preceded by novel [NN], familiar preceded by novel [NF), self preceded by familiar [FS), novel preceded by familiar [FN], and familiar preceded by familiar [FF]) with 20 trials per condition and block. Each regressor was accompanied by a parametric modulator reflecting subjective value from the respective agent’s perspective. This value was calculated based on a trial-by-trial estimate of the subject’s current belief about their partners’ discount rate k. Furthermore, we defined one choice regressor per agent and block indexing the time at which subjects indicated their decision on choice trials and received feedback. Each was accompanied by a parametric regressor corresponding to the surprise subjects experienced as they observed the partner’s choice. Button presses were included as a regressor of no interest. Because of the sensitivity of the BOLD signal in the OFC region to subject motion and physiological noise, we included six motion regressors obtained during realignment as well as ten regressors for cardiac phase, six for respiratory phase, and one for respiratory volume extracted with an in-house-developed Matlab toolbox as nuisance regressors (Hutton et al., 2011). Blocks were modeled separately within the GLM.

To detect areas showing adaptation to repeated agents as depicted in Figure 2A, we used the contrast ([agentprecededbydifferentagent]−[agentprecededbysameagent]) (i.e., ([SN+SF+NS+NF+FS+FN]−2[SS+FF+NN])). To test for areas displaying greater increases in suppression between self and the novel other compared to between self and familiar other (Figure 2B), we defined the following contrast: $\left({\left[SN+NS\right]}_{block1}-{\left[SN+NS\right]}_{block3}\right)-\left({\left[SF+FS\right]}_{block1}-{\left[SF+FS\right]}_{block3}\right)$. To test for greater increases in suppression between self and novel other than between novel other and familiar other, we defined a contrast as follows: $\left({\left[SN+NS\right]}_{block1}+{\left[SN+NS\right]}_{block3}\right)-\left({\left[NF+FN\right]}_{block1}-{\left[NF+FN\right]}_{block3}\right)$.

The contrast images of all subjects from the first level were analyzed as a second-level random effects analysis. Results are reported at a cluster-defining threshold of p < 0.01 uncorrected combined with a FWE-corrected significance level of p < 0.05.

We performed a jack-knife procedure from the mPFC ROI (Figure 2B) to extract parameter estimates from this region without biasing the selection. To this end, we extracted parameter estimates for each subject from an ROI defined according to all other subjects (threshold at p < 0.01 uncorrected). This signal was used to perform all analyses depicted in Figures 2–4 and S3A.

We performed partial correlations to control for correlations between shift_self→novel,scan_ and shift_fam→novel,scan_ in our analysis of the relationship of a behavioral shift effect and neural plasticity. This removes the shift of the familiar other toward the novel other from the subjects’ own discount rate shifts and the neural plasticity index [SN − SF]_1−3_ (Figure 3A) and the shift of self toward the novel other from the familiar other’s shift toward the novel other and the neural plasticity index (Figure 3B). We also estimated a linear regression model on the same data with shift_self→novel,scan_ and shift_fam→novel,scan_ as independent variables and [SN − SF]_1−3_ as the dependent variable. The relationship between shift_self→novel,scan_ and [SN − SF]_1−3_ was directly contrasted with the relationship between shift_fam→novel,scan_ and [SN − SF]_1−3_.

To test for the influence of surprise on mPFC plasticity, we defined a contrast assessing BOLD correlate of the surprise subjects experienced as they got feedback about the novel and the familiar partners’ choices. This contrast revealed activity in ACC, in bilateral insula and dorsal striatum (Figure 4A; note that insula and striatal activity did not survive cluster-based FEW thresholding). To identify the surprise experienced when learning about the novel other, parameter estimates were then extracted from these ROIs for the novel other’s choices only. This surprise measure in the striatum was correlated with subjects’ shift in discount rate (Figure 4B) and the plasticity measure [SN − SF]_1−3_ extracted from the mPFC ROI (Figure 4C).

We used the Mediation and Moderation Toolbox (Atlas et al., 2010; Wager et al., 2008) to perform a mediation analysis on this surprise signal, our plasticity measure, and the discount rate shift.

To test the specificity of adaptation effects, we analyzed repetition suppression effects in visual regions. We defined an ROI from a contrast identifying a main effect to any visual event, averaged across all blocks, and performed the same analyses as for the mPFC ROI (thresholded at p < 0.0001 uncorrected; Figure S2).

## Supplemental Information

Document S1. Figures S1–S5 and Supplemental Experimental Procedures

Document S2. Article plus Supplemental Information
