# Supplementary material for: Learning-Induced Plasticity in Medial Prefrontal Cortex Predicts Preference Malleability
Source: Neuron. 2015 Jan 21;85(2):418–28. doi: 10.1016/j.neuron.2014.12.033 (PMC4306543; doi:10.1016/j.neuron.2014.12.033)
Supplement: Document S1. Figures S1–S5 and Supplemental Experimental Procedures [file mmc1.pdf]

Neuron, Volume *85*

Supplemental Information

Learning-Induced Plasticity

in Medial Prefrontal Cortex

Predicts Preference Malleability

Mona M. Garvert, Michael Moutoussis, Zeb Kurth-Nelson, Timothy E.J. Behrens, and Raymond J. Dolan

Supplemental Figure S1

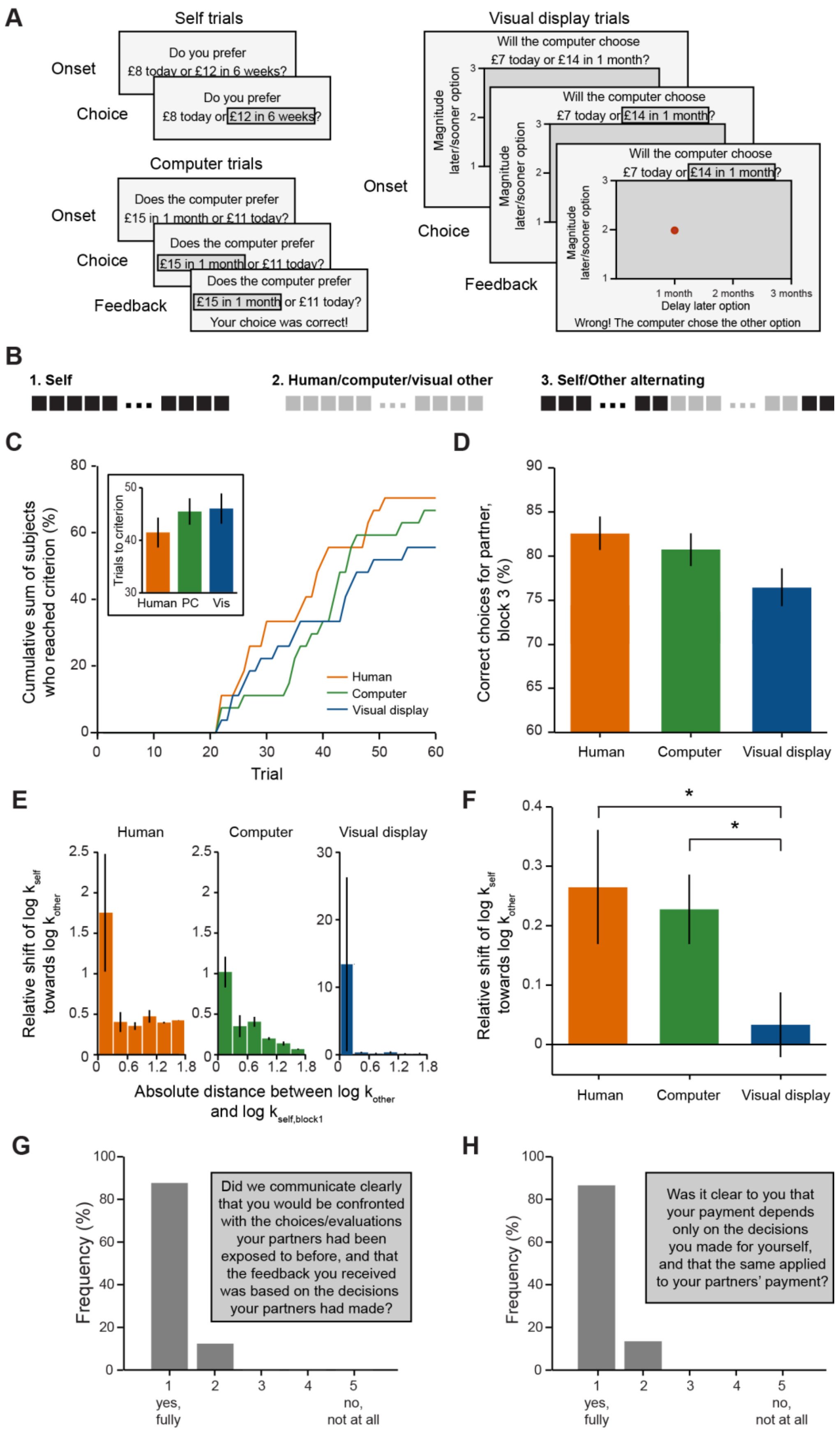

Figure S1 (related to Figure 1). Learning the preferences of another and its consequences for behaviour

(A) On self trials, subjects chose for themselves between an amount of money available on the same day and a larger amount of money available after a delay. On human and computer partner trials, subjects made these kinds of choices on behalf of their confederate. On visual display trials, the choice pair was presented on a 2D grid. Subjects were instructed to choose according to their belief about the orientation of an imaginary isoprobability line (see Supplemental Experimental Procedures for details). After each human partner, computer and visual display trial, feedback indicated whether a choice was correct.

(B) Experimental structure: Block 1 consisted of self choice trials alone, block 2 consisted of other choice trials alone and block 3 consisted of alternating short blocks of 10 choice trials per agent (self or other, see Figure 1B).

(C) Block 2 terminated after 17 correct choices for the confederate within a sliding window of 20 consecutive trials or a maximum of 60 trials. The graph depicts the cumulative sum of subjects who terminated after a given number of trials. The inset shows the mean number of trials to criterion for the different groups. The average number of trials subjects needed to reach criterion in block 2 did not differ between groups ($F_{2,78} = 0.82$, $P = 0.44$).

(D) Correct choices for the confederate in block 3 did not differ between groups ($F_{2,78} = 2.55$, $P = 0.08$). This suggests that subjects in all three groups learnt the other's discount rate equally well.

(E) Discount rate shift ($\text{shift} = \frac{\log \boldsymbol{k}_{\boldsymbol{self,block}\,3} - \log \boldsymbol{k}_{\boldsymbol{self,block1}}}{\log \boldsymbol{k}_{\boldsymbol{other,block}\,2} - \log \boldsymbol{k}_{\boldsymbol{self,block1}}}$) binned according to the distance between $|\log k_{other,block2} – \log k_{self,block1}|$. As shift estimates were inflated for $|\log k_{other,block2} - \log k_{self,block1}| \leq 0.3$, subjects who estimated the other's discount rate to be within that range were excluded from all discount rate shift analyses in the behavioural and the fMRI experiment.

(F) Shift of subjects' own discount rate in the direction of the partner's discount rate relative to the distance between $\log k_{self,block1}$ and $\log k_{other,block2}$. Subjects in the human and the computer, but not the visual display condition shifted towards the preferences of their partner ($t_{21} = 3.06$, $P = 0.006$, $t_{23} = 3.66$, $P = 0.001$ and $t_{24} = 0.61$, $P = 0.55$ respectively). A one-way ANOVA revealed that the difference in shift towards the other's preferences differed between experimental groups ($F_{2,68} = 3.5$, $P = 0.04$). Post-hoc t-tests attributed this difference to a smaller shift in the visual display group: Both subjects in the human and in the computer partner group displayed a stronger shift in discount rate towards the other than subjects in the visual display group ($t_{45} = 2.37$, $P = 0.02$ and $t_{47} = 2.25$, $P = 0.03$). There was no difference in shift towards the partner for the human versus the computer partner group ($t_{44} = 0.61$, $P = 0.5$). Subjects' estimate of the novel other's discount rate did not change from block 2 to block 3 in any of the three conditions (relative shift of other's discount rate

calculated as $\frac{\log k_{other,block\,3} - \log k_{other,block\,2}}{\log k_{self,block\,1} - \log k_{other,block\,2}}$, human partner: $t_{21} = 0.99$, $P = 0.33$, computer partner: $t_{23} = -1.75$, $P = 0.09$ and visual display: $t_{24} = -0.01$, $P = 0.99$). This confirms that discount rates for self and other are not converging. Instead the change in discount rate corresponds to a selective shift of subjects' own discount rate towards the discount rate of the other. Note we cannot rule out differences in terms of attention, working memory or other factors that prevent the update of one's own preferences for the visual display condition. Since stimuli and actions were the same as in the human and the computer partner condition, however, we can rule out that the behavioural shift in the other experimental conditions is due to simple stimulus- or action-reinforcement.

(G) After finishing the experiment, subjects completed a debriefing questionnaire designed to assess the credibility of the experimental design. Illustrated is the percentage of subjects answering 1 (yes, fully) to 5 (no, not at all) in response to the following question: Did we communicate clearly that you would be confronted with the choices and evaluations your partners had been exposed to before, and the feedback you received was based on the decisions your partners had made?

(H) Percentage of subjects answering 1 (yes, fully) to 5 (no, not at all) to the following question: Was it clear to you that your payment depends only on the decisions you made for yourself, and that the same applied to your partners' payment?

Data are represented as mean ± SEM. All post-hoc tests were Bonferroni-corrected for multiple comparisons.

Supplemental Figure S2

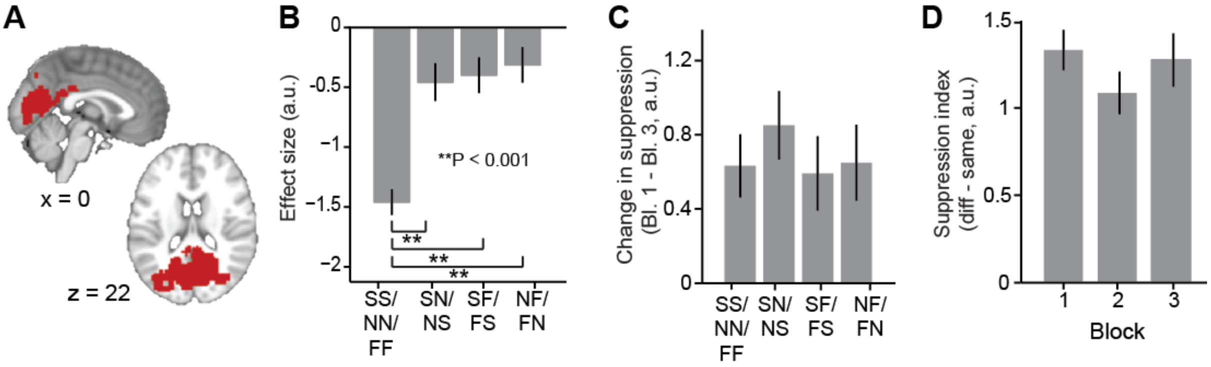

Figure S2 (related to Figure 2). Repetition suppression in visual areas

(A) Region of interest used to interrogate plasticity effects in visual regions (thresholded at $P < 0.001$ uncorrected for visualization). The region of interest was defined from a contrast indexing a main effect to any visual event in all three blocks.

(B) Visual areas displayed significantly less activity when the agent from a preceding trial was repeated than when a different agent preceded a trial ($F_{3,104} = 14.25$, $P < 0.0001$, block 1 only).

(C) Suppression increased over blocks with no difference between conditions ($F_{3,104} = 0.37$, $P = 0.78$).

(D) The difference in mean activity on same-agent-preceding trials versus different-agent-preceding trials did not change over blocks ($F_{2,78} = 0.32$, $P = 0.73$).

SS: self-preceded-by-self, NN: novel-preceded-by-novel, FF: familiar-preceded-by-familiar, SN: novel-preceded-by-self, NS: self-preceded-by-novel, SF: familiar-preceded-by-self, FS: self-preceded-by-familiar, NF: familiar-preceded-by-novel, FN: novel-preceded-by-familiar. a.u., arbitrary units.

Supplemental Figure S3

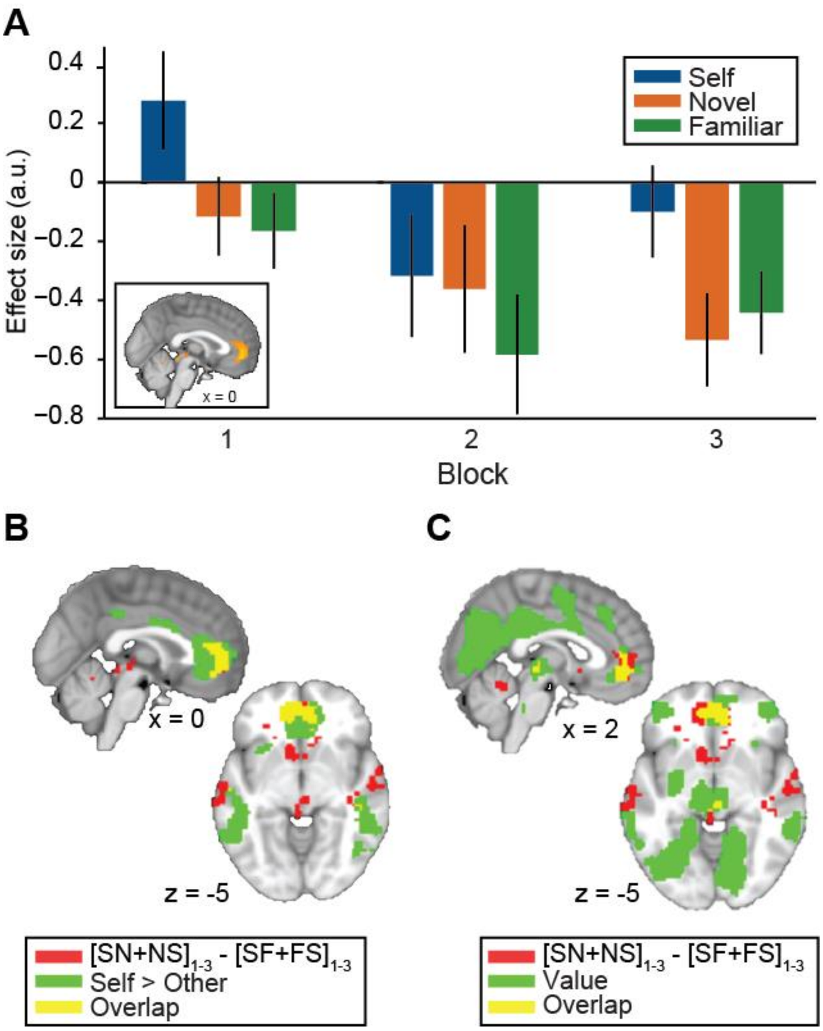

Figure S3 (related to Figure 2). MPFC activity for self, other and value

(A) Activity for self, novel and familiar other over blocks in the medial prefrontal cortex. A repeated measures ANOVA with within-subject factors "block" and "agent" showed that activity differed over blocks ($F(2,52) = 13.21$, $P < 0.001$) and between agents ($F(2,52) = 4.19$, $P = 0.05$). Furthermore, we found a block x agent interaction ($F(4,104) = 3.09$, $P = 0.02$). Post-hoc tests revealed that activity in block 1 was different from activity in blocks 2 and 3 ($P = 0.005$ and $P < 0.001$, respectively), but activity in blocks 2 and 3 did not differ ($P = 0.88$). Activity between familiar and novel other did not differ ($P = 1.0$), suggesting that the plasticity effect we report cannot be explained by differences in novelty/familiarity for the two agents. Parameter estimates were extracted from ROI shown in Figure 2B (see inset).

(B) Overlap between self > other contrast and mPFC plasticity. Mean activity on self trials was higher than on other trials in left lateral parietal cortex ($P < 0.001$, peak $t_{26} = 6.26$, peak [-39, -79, 34]) and in the mPFC ($P < 0.001$, peak $t_{26} = 6.08$, peak [9, 41, -5], thresholded at P

< 0.01 uncorrected for visualization). Activity in the mPFC overlapped with the region showing an increase in suppression between self and novel, controlled for by an increase in suppression between self and familiar as depicted in Figure 2B. The opposite contrast (other > self) only revealed activity in the visual cortex ($P < 0.001$, peak $t_{26} = 8.83$, peak [0, -94, 7], not depicted).

(C) Subjective value coding on probe trials and mPFC plasticity. Subjective value was encoded in left primary motor cortex ($P < 0.001$, peak $t_{26} = 9.54$, peak [-36, -25, 55]), in right parietal cortex ($P < 0.001$, peak $t_{26} = 5.04$, peak [54 -16 22]), in Brodmann area 10 ($P = 0.031$, peak $t_{26} = 4.37$, peak [-18, 62,7]) and in mPFC ($P = 0.055$, peak $t_{26} = 4.26$, peak [9, 44, 10], thresholded at $P < 0.01$ uncorrected for visualization). Activity in the mPFC overlapped with the region showing an increase in suppression between self and novel, controlled for by an increase in suppression between self and familiar as depicted in Figure 2B.

Results are reported at a cluster-defining threshold of $P < 0.01$ uncorrected combined with a family-wise-error (FWE) corrected significance level of $P < 0.05$. All post-hoc tests were Bonferroni-corrected for multiple comparisons. a.u., arbitrary units.

Supplemental Figure S4

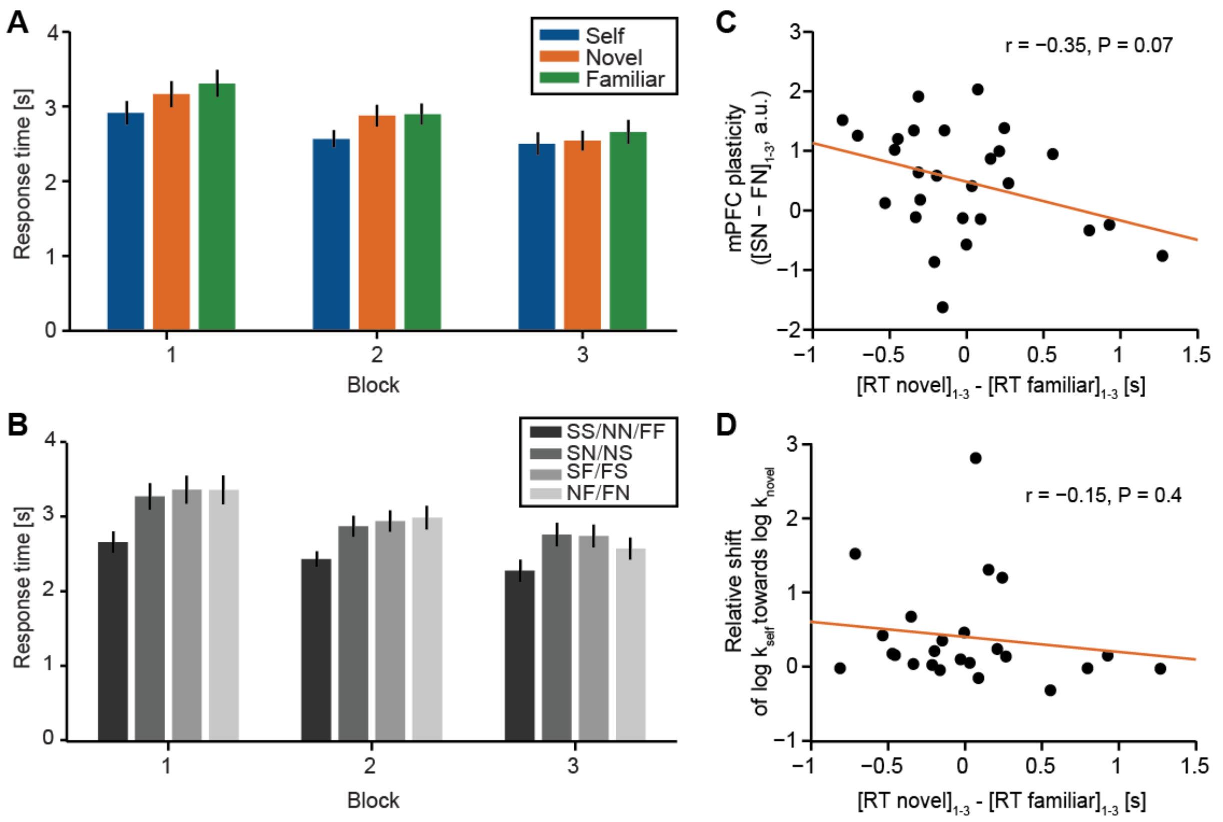

Figure S4 (related to Figure 2). Response time analyses

(A) Response times for self, novel and familiar other probe trials over blocks. A repeated measures ANOVA with within-subject factors “block” and “agent” showed different response times between blocks ($F(2,52) = 21.28$, $P < 0.001$), agents ($F(2,52) = 19.8$, $P < 0.001$) as well as a block x agent interaction ($F(4,104) = 2.88$, $P = 0.03$). Post-hoc tests reveal differences between all blocks (blocks 1/2: $P = 0.002$, blocks 1/3: $P < 0.001$, blocks 2/3: $P = 0.04$). Furthermore, subjects respond faster for self than for other ($P < 0.001$ for self/novel and self/familiar). Importantly, we found no significant difference in response times for novel and familiar other ($P = 0.29$), confirming that there is no novelty/familiarity effect.

(B) To test for behavioural suppression effects that are in line with the neural suppression effects, we performed a repeated measures ANOVA with Greenhouse-Geisser correction with within subject factors “block” and “suppression condition” (SS/NN/FF, SN/NS, SF/FS, FN/NF) on subjects’ response times on probe trials. We found a main effect of block ($F(1.442,37.500) = 21.3$, $P < 0.001$), a main effect of condition ($F(2.331,60.609) = 41.7$, $P < 0.001$), and a block x condition interaction ($F(4.571,118.859) = 4.5$, $P = 0.001$). Post-hoc paired tests revealed that SS/NN/FF differed from all other conditions ($P < 0.001$), but SN/NS, SF/FS and FN/NF did not differ from each other (all comparisons $P = 1.0$). This

emphasizes that the neural suppression effects between self and novel, and between self and familiar, respectively, cannot simply be explained by faster processing speed.

(C) Correlation between response time facilitation for the novel other (RT novel block 1 – RT novel block 3) – (RT familiar block 1 – RT familiar block 3) and $[SN\text{-}SF]_{1\text{-}3}$ plasticity effect in mPFC (ROI from Figure 2B). Response time facilitation, a crude index of increasing familiarity, shows a trend towards a negative correlation with the neural plasticity effect ($R = -0.35$, $P = 0.07$). This is in line with an observation that the opposite of familiarity, namely surprise, is a better predictor of mPFC plasticity.

(D) Response time facilitation for the novel other does not correlate with the behavioural discount rate shift of self towards the novel other ($R = -0.15$, $P = 0.4$).

All post-hoc tests were Bonferroni-corrected for multiple comparisons.

SS: self-preceded-by-self, NN: novel-preceded-by-novel, FF: familiar-preceded-by-familiar, SN: novel-preceded-by-self, NS: self-preceded-by-novel, SF: familiar-preceded-by-self, FS: self-preceded-by-familiar, NF: familiar-preceded-by-novel, FN: novel-preceded-by-familiar. a.u., arbitrary units.

Supplemental Figure S5

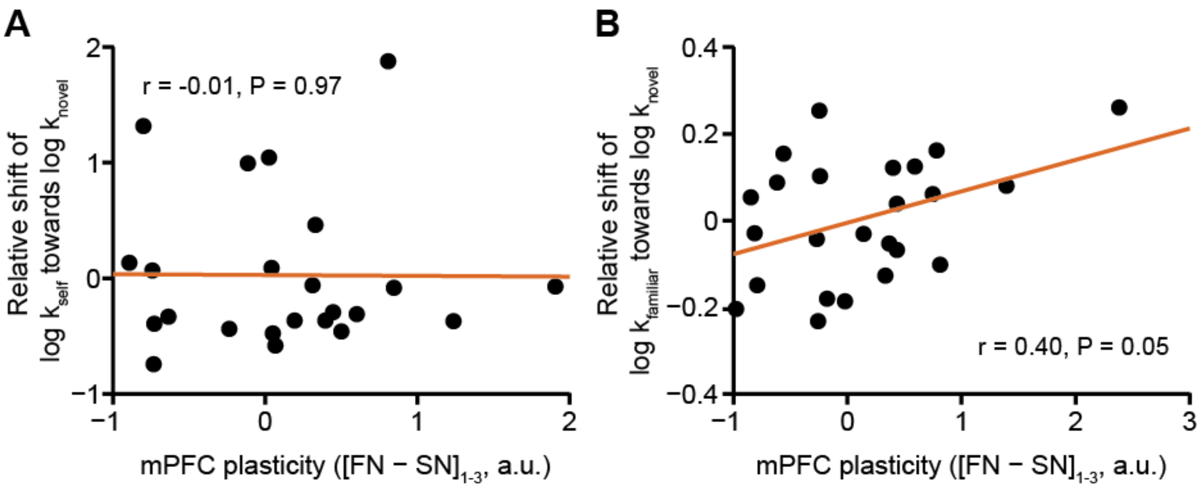

Figure S5 (related to Figure 3). Relationship between $[FN\text{-}SN]_{1\text{-}3}$ plasticity and shift in discount rate

(A) Partial correlation between the change in suppression between familiar and novel other controlled for by the change in suppression between self and novel other and the shift of subjects' own discount rate towards the novel other.

(B) Partial correlation between the change in suppression between self and novel other controlled for by the change in suppression between self and familiar other and the shift of subjects' estimate of the familiar other's discount rate towards the novel other.

Parameter estimates in A and B were extracted from the mPFC ROI shown in Figure 2B. To account for the correlation between subjects' own shift in discount rate and the shift in their estimate of the familiar other's discount rate, we performed partial correlations, i.e. the familiar shift was removed from both signals in A and the self shift was removed from both signals in B. These analyses indicate that the change of familiar-to-novel suppression versus the change in self-to-novel suppression predicted a shift in subjects' estimate of the familiar other's discount rate (partial correlation, $r = 0.40$, $P = 0.05$, Figure S5B) but not the shift in subjects' own discount rate (partial correlation, $r = -0.01$, $P = 0.97$, Figure S5A). This emphasizes the relationship between increasing neuronal similarity between two agents' value representations and increasing behavioural similarity, as depicted in Figure 3. However, note that these data are merely suggestive, as removal of the rightmost data point in Figure S5B affects the significance of the result.

a.u., arbitrary units.

Supplemental Experimental Procedures

Behavioural and fMRI task

Pairs of subjects were introduced to each other as partners before the experiment and instructed simultaneously, but performed the task in separate rooms. Both subjects made a series of choices between a smaller amount paid on the same day and a larger amount paid later (Figure S1A). The amounts varied between £1 and £20 and the delay was *tomorrow, 1 week, 2 weeks, 4 weeks, 6 weeks, 2 months,* or *3 months*. The two options were presented simultaneously and the location of the immediate and delayed option on the screen was randomized. Subjects chose by pressing a button corresponding to the location of their preferred option without any time constraint.

In block 2, subjects were instructed that they would be exposed to their partner's options from block 1 and were instructed to reproduce the partner´s decisions. Choices were correct if they corresponded to the decision that would be preferred by a hyperbolic discounter with the discount rate used to generate the decisions (see below for details).

One of the outcomes (two in the fMRI experiment) chosen by the subject for themselves was randomly selected at the end of the experiment and transferred to their bank account after the respective delay. Subjects knew that their monetary outcome depended on their own choices alone and that the choices they made for the other were not communicated to the partner and did not have any consequences for either subject (Figure S1G, H).

In the fMRI experiment, a set of probe trials was added to the experiment. The combinations of amount and delay on probe trials were drawn randomly from the same set as the options presented in choice trials. Subjects were instructed to choose according to their own preferences ("self" trials) or according to the choice their partners had made when they had participated in the same experiment previously ("other" trials).

Subjects learned the preferences of a second partner ('familiar other') before the scan (Figure 1E, top). In this pre-scan session, subjects performed one block of choices and evaluations for self before and after a block of choices for the partner. Each pre-training

block contained 48 choice trials as well as 16 randomly interleaved probe trials to familiarize subjects with this trial type. Each of the three experimental blocks in the scanner consisted of 197 probe trials for self, novel other and familiar other and 16 short interleaved blocks of one choice trial per agent (Figure 1E, bottom).

Estimation of discount rates

We estimated subjects' discount rates separately for each experimental block by fitting a hyperbolic model to their choices (Rachlin et al., 1991). On every trial, we calculated the subjective value of both options as

(1) $V = \frac{M}{1+kD}$

where V is the subjective value, M is the magnitude, D is the delay and k is a subject-specific discount rate that quantifies the devaluation of future rewards. When k = 0, subjects do not discount future rewards and base their valuation of an option purely on its magnitude. As k grows, subjects discount future rewards more and more steeply. Since the delay of the smaller option was always 0 (*today*), the subjective value of the smaller option (V_{SS}) always corresponded to its magnitude. We used a softmax function to transform the difference in subjective value between the two choice options (V_{LL}-V_{SS}) on each trial into choice probabilities:

(2) $P(LL) = \frac{1}{1+e^{-\beta(V_{LL}-V_{SS})}}$

where β is a subject-specific inverse temperature parameter that characterizes non-systematic deviations around the indifference point. We then applied maximum likelihood estimation across trials to optimize k and β. Parameter estimates were constrained such that -4 < log k < 0 and -1 < log β < 1. Note that all discount rate analyses in this paper were performed in $\log_{10}$ space, transforming typical discount rates of [0.0001 – 0] to the range [-4 – 0].

Note a similar approach also provided us with a trial-by-trial estimate of subjects' discount rates and temperature parameters. We started off with a uniform prior, which was updated

on each trial according to Bayes rule: the posterior was calculated as the likelihood of a subject's choice given the parameters k and β weighed by the prior. This posterior was updated on subsequent trials according to the same measure. This trial-by-trial discount rate estimate could then be used to calculate the subjective value of probe trials according to Eq. 1 as well as the surprise subjects' experienced surprise on choice trials as outlined in the Experimental Procedures. According to the same procedure, we extracted trial-by-trial estimates of the novel and familiar others' discount rate from choices for their partners and computed the subjective value on partner trials based on these estimations (Eq. 1). This trial-by-trial value signal was used as a parametric modulator on probe trials in our fMRI GLM. Furthermore, we also estimated the surprise subjects experienced when comparing the choice they predicted a partner would make to the choice they actually observed their partners make. To this end, we applied the same procedure as outlined above, but we now used subjects' trial-by-trial belief about the familiar and the novel other's discount rate to estimate surprise. This measure was used as a parametric modulator in a second independent GLM which was otherwise set up exactly like the first GLM to test whether this different surprise measure would also be represented in the brain.

Optimization of choice pairs in the behavioural and scan experiment

In order to accurately estimate subjects' shift in discount rate, we needed an efficient and precise estimate of subjects' discount rates. To optimize choice pairs for this purpose, we alternated between two option generation methods on choice trials for self. In method 1, we first generated all possible pairs of amounts and delays and selected a subset of n/2 trials (n = total number of trials in a block) that best matched the indifference points of n/2 hypothetical subjects whose discount rates log k were evenly distributed between [-4:0] in $\log_{10}$ space (Nicolle et al., 2012). This procedure allowed for an efficient, but relatively imprecise estimate of subjects' discount rates.

To increase the precision of our estimate of subjects' discount rates, we alternated the thus generated trials with choices generated according to a second method, which was adaptive

in nature and based on the same Bayesian framework outlined above for estimating subjects' discount rates on a trial-by-trial basis. Here, the population distribution of log k with a mean of -2 and a standard deviation of 1 was taken as a prior belief about an individual's log k. Every time the subject made a decision, this belief distribution about their log k was updated using Bayes rule. Questions were then generated to probe our estimate of subjects' indifference point (where both options are equally preferred), which we estimated to correspond to the expected value of the current posterior. The thus generated choices were more informative about the subjects' exact discount rate than many of the other choice trials such that this procedure gave us a more precise estimate of the subjects' exact discount rate. We validated the adaptive Bayesian method against the standard non-adaptive method and found that the adaptive method produced similar results as the non-adaptive method, using fewer trials. However, data from both methods were included in the final analysis to maximize power.

Choice pairs on "other" trials, were selected as a set of trials that best matched the indifference points of 60 (block 2) vs. 100 (block 3) hypothetical subjects whose discount rates were evenly distributed across the range centred on the other's discount rate [log k_{other}-1; log k_{other}+1]. This ensured that the number of immediate and delayed choices the subject made for the partner was approximately equal.

In the fMRI experiment, choice trials were not centred on the other's discount rate, because this would have generated different kinds of choices for the novel and the familiar other. Instead, choice pairs on "other" trials in the fMRI experiment were selected as a set of trials that best matched the indifference points of 48 hypothetical subjects whose discount rates were evenly distributed across the range [-4; 0]. Choice pairs for the other were generated according to the non-adaptive method only.

Subjects in control experiment

54 volunteers (mean age ± std: 23.0 ± 7.9, 31 females) participated in the two control experiments. 27 were randomly assigned to a computer and a visual choice task condition,

respectively. There were no significant differences in age ($F_{2,78}$ = 0.71, P = 0.49) or gender ($F_{2,78}$ = 1.04, P = 0.36) between the human partner, the computer partner, and the visual display group. All subjects were neurologically and psychiatrically healthy. The study took place at the Wellcome Trust Centre for Neuroimaging in London, UK. The experimental procedure was approved by the University College London Hospitals Ethics Committee and written informed consent was obtained from all subjects.

Computer partner and visual display conditions

Subjects in the computer partner condition were told that a computer programme was trained to make decisions according to a specific strategy, while all other experimental settings were the same as in the human partner group.

Subjects assigned to the visual display condition learned a discount rate without engaging in any form of simulation. Instead, subjects were presented with a geometric depiction of a given choice on the screen (Figure S1A, right) where the x-axis of the rectangle represented the delay of the delayed option and the y-axis represented the ratio of magnitudes for the delayed and the immediate options (M_{LL}/M_{SS}). Subjects were told that the computer was programmed to choose one of the two options according to the location of the dot relative to an isoprobability line, which they had to learn based on the feedback they received after each choice. In fact, decisions in this version were generated according to the same method as for human and computer partner groups.

The orientation of the isoprobability line that subjects had to learn was determined by the discount rate k, with larger discount rates corresponding to a steeper line. This follows from comparing choices for which the value of the immediate and the delayed option are equal:

(3) $$V_{SS} = V_{LL} \Leftrightarrow M_{SS} = \frac{M_{LL}}{1+kD_{LL}} \quad \Leftrightarrow {M_{LL}}/{M_{SS}} = kD_{LL} + 1$$

Subjects were instructed that dots above the line correspond to choosing the delayed, and dots below the line correspond to choosing the immediate option. Again, choice was

translated into probabilities with a softmax function (Eq. 2) such that choices were noisy and this was communicated to the subjects.

Exclusion criterion for shift analyses

Some subjects estimated their confederate's discount rate to be very similar to their own. As a result even small shifts of subjects' own discount rate was substantially inflated when comparing it to the distance log $k_{self,block1}$ – log $k_{other,block2}$. Therefore, subjects with an absolute distance $|\log k_{other,block2} - \log k_{self,block1}| \leq 0.3$ were excluded from analyses. This value seemed to constitute a tipping point in all three experimental groups with strongly overrated shifts in discount rate for subjects with $|\log k_{other,block2} - \log k_{self,block1}| \leq 0.3$ (Figure S1F). According to this criterion, we excluded 5 subjects in the human partner condition, 3 subjects in the computer partner condition and 2 subjects in the visual display condition.

To test the robustness of our procedure to the particular threshold we chose, we examined the results for a threshold of 0.5. This excluded 12 subjects in the human partner condition, 4 subjects in the computer partner condition and 2 subjects in the visual display condition. Importantly, we still found subjects in the human and computer, but not the visual display group to shift towards the preferences of their partners: ($t_{14} = 2.76$, $P = 0.02$, $t_{22} = 3.89$, $P = 0.001$ and $t_{24} = 0.61$, $P = 0.5$, respectively) with a significant difference between groups ($F_{2,60} = 3.7$, $P = 0.03$).

Scan procedure, fMRI data acquisition and pre-processing

Visual stimuli were projected onto a screen via a computer monitor. Subjects indicated their choice using an MRI-compatible button box. Stimuli were presented for a minimum duration of 3 to 5 seconds (jittered) or until subjects indicated their decision. MRI data was acquired using a 32-channel head coil on a 3 Tesla Allegra scanner (Siemens, Erlangen, Germany). A special sequence was used to acquire T2*-weighted echo-planar images (EPI) to minimize susceptibility related artefacts in the ventral prefrontal cortex (Weiskopf et al. 2006): 43 transverse slices (ascending order) of 2 mm thickness with 1-mm gap and in-plane

resolution of 3x3 mm, a repetition time of 3.01 s and an echo time of 70 ms were collected. Slices were tilted by 30° relative to the rostro-caudal axis and a local z-shim with a moment of -0.4 mT/m was applied to the OFC region. The first five volumes of each block were discarded to allow for equilibration. A T1-weighted anatomical scan with 1x1x1 mm resolution was acquired at the end of the session in order to spatially normalize the EPIs. In addition, a whole-brain fieldmap with dual echo-time images (TE1 = 10 ms, TE2 = 14.76 ms, resolution 3x3x3 mm) was obtained to correct for geometric distortions induced in the EPIs at high field strength.

We used SPM8 for image pre-processing and data analysis (Wellcome Trust Centre for Neuroimaging, London UK). We corrected for signal bias, co-registered functional scans to the first volume in the sequence and corrected for distortions using the fieldmap. Data were spatially normalized to a standard EPI template and smoothed using a 8 mm full-width at half maximum Gaussian kernel.

Physiological noise in the GLM

To reduce the contribution of physiological noise to the BOLD signal (Hutton et al. 2011), the cardiac pulse was recorded using an MRI compatible pulse oximeter (Model 8600 F0, Nonin Medical Inc., Plymouth, MN, USA) and thorax movement was monitored using a custom-made pneumatic belt positioned around the abdomen. The pneumatic pressure was converted into an analogue voltage signal using a pressure transducer (Honeywell International Inc., Morristown, NJ, USA) before digitization.

Models for cardiac and respiratory phase and their aliased harmonics were based on RETROICOR (Glover et al., 2000); the model for respiratory volume changes was based on (Birn et al., 2006). Slice 15 was used as a reference slice for modelling fluctuations arising from cardiac phase because of its proximity to the OFC (Hutton et al., 2011).

Mediation analysis

We used the used the Mediation and Moderation Toolbox (Atlas et al., 2010; Wager et al., 2008) to perform a single-level mediation analysis. To test whether the mPFC plasticity mediates the effect of striatal surprise on behavioural shift, we first extracted each individual's parameter estimate from the striatal ROI encoding surprise. The mediator corresponded to each subject's plasticity index $[SN\text{-}SF]_{1\text{-}3}$ computed from parameter estimates extracted from the mPFC ROI. The outcome variable was defined as a subject's relative shift in discount rate towards the novel other. The relationship between striatal surprise and behavioural shift controlling for the mPFC effect is referred to as path "c". We also estimated the relationship between striatal surprise and mPFC plasticity (path "a") as well as between mPFC plasticity and behavioural shift (path "b"). This last path "b" is controlled for striatal surprise, such that paths "a" and "b" correspond to two separable processes contributing to the behavioural effect. A mediation test (path "ab") examines whether the mediator (mPFC plasticity) explains a significant amount of the covariance between striatal surprise and behavioural shift. We determined two-tailed uncorrected P values from the bootstrap confidence intervals for the path coefficients (Atlas et al., 2010).

Supplemental References

Birn, R.M., Diamond, J.B., Smith, M.A., and Bandettini, P.A. (2006). Separating respiratory-variation-related fluctuations from neuronal-activity-related fluctuations in fMRI. NeuroImage *31*, 1536–1548.

Glover, G.H., Li, T.Q., and Ress, D. (2000). Image-based method for retrospective correction of physiological motion effects in fMRI: RETROICOR. Magn. Reson. Med. *44*, 162–167.
