# Supplementary material for: Learning-Induced Plasticity in Medial Prefrontal Cortex Predicts Preference Malleability
Source: Neuron. 2015 Jan 21;85(2):418–28. doi: 10.1016/j.neuron.2014.12.033 (PMC4306543; doi:10.1016/j.neuron.2014.12.033)
Supplement: Document S2. Article plus Supplemental Information [file mmc2.pdf]

Neuron

Article

Learning-Induced Plasticity in Medial Prefrontal Cortex Predicts Preference Malleability

Highlights

- Learning the values of another causes plasticity in a mPFC value representation
- This plasticity predicts how much subjects' own preferences change
- Plasticity is explained by a striatal surprise signal
- Value coding in mPFC occurs independently of the agent for whom a decision is made

Authors

Mona M. Garvert, Michael Moutoussis, ..., Timothy E.J. Behrens, Raymond J. Dolan

Correspondence

mona.garvert.11@ucl.ac.uk

In Brief

Garvert et al. demonstrate that learning the preferences of another person increases the similarity between neural value representations for self and other. This plasticity in medial prefrontal cortex predicts how much one's own preferences shift toward those of the other.

Garvert et al., 2015, Neuron *85*, 418–428
January 21, 2015 ©2015 The Authors
http://dx.doi.org/10.1016/j.neuron.2014.12.033

Cell Press
Neuron
Article

Learning-Induced Plasticity in Medial Prefrontal Cortex Predicts Preference Malleability

Mona M. Garvert,[1,*] Michael Moutoussis,[1] Zeb Kurth-Nelson,[1,2] Timothy E.J. Behrens,[1,3] and Raymond J. Dolan[1,2]
[1]Wellcome Trust Centre for Neuroimaging, Institute of Neurology, University College London, London WC1N 3BG, UK
[2]Max Planck UCL Centre for Computational Psychiatry and Ageing Research, Russell Square House, 10-12 Russell Square, London WC1B 5EH, UK
[3]Oxford Centre for Functional MRI of the Brain, Nuffield Department of Clinical Neurosciences, University of Oxford, John Radcliffe Hospital, Oxford OX3 9D, UK
*Correspondence: mona.garvert.11@ucl.ac.uk
http://dx.doi.org/10.1016/j.neuron.2014.12.033
This is an open access article under the CC BY license (http://creativecommons.org/licenses/by/3.0/).

SUMMARY

Learning induces plasticity in neuronal networks. As neuronal populations contribute to multiple representations, we reasoned plasticity in one representation might influence others. We used human fMRI repetition suppression to show that plasticity induced by learning another individual's values impacts upon a value representation for oneself in medial prefrontal cortex (mPFC), a plasticity also evident behaviorally in a preference shift. We show this plasticity is driven by a striatal "prediction error," signaling the discrepancy between the other's choice and a subject's own preferences. Thus, our data highlight that mPFC encodes agent-independent representations of subjective value, such that prediction errors simultaneously update multiple agents' value representations. As the resulting change in representational similarity predicts interindividual differences in the malleability of subjective preferences, our findings shed mechanistic light on complex human processes such as the powerful influence of social interaction on beliefs and preferences.

INTRODUCTION

Information in the brain is encoded within distributed neuronal populations such that individual neurons typically support more than one representation or computation. Neurons in medial prefrontal cortex (mPFC), for example, perform self-referential as well as social value computations (Jenkins et al., 2008; Nicolle et al., 2012; Suzuki et al., 2012). Whereas it is traditionally suggested that computations for self and other are performed within separate populations of neurons (D'Argembeau et al., 2007; Denny et al., 2012), recent work suggests a functional organization within this region does not neatly conform to such a distinction by agent. Instead, value computations on behalf of any individual can be realized by the same circuitry (Nicolle et al., 2012), and the neural code depends only on the subjective value of an offer. In light of this, we conjectured that multiple value computations might be updated simultaneously if plasticity is introduced into this circuitry.

The contribution of overlapping neural circuitry to distinct computations has previously been demonstrated during delegated inter-temporal choice (Nicolle et al., 2012). In inter-temporal choice paradigms, subjects reveal their preferences for larger reward delivered later versus smaller reward that arrive sooner. Choice in this context is quantified by a "temporal discount rate" (Myerson and Green, 1995), believed to index forms of behavioral impulsivity (Evenden, 1999; Robbins et al., 2012) and an ability to imagine future outcomes (Ersner-Hershfield et al., 2009; Mitchell et al., 2011; Peters and Büchel, 2010). When subjects are asked to make such inter-temporal choices on behalf of another individual ("delegated inter-temporal choice"), they rapidly learn the confederate's discount rate (Nicolle et al., 2012). This adaptability depends on the medial prefrontal cortex, where a neural circuitry used to compute a subject's own values also computes those of a confederate, enabling rapid switches between the two computations (Nicolle et al., 2012).

We reasoned that if the same circuitry in the mPFC computes the value of a delayed offer irrespective of agents, plastic changes necessary to learn a new partner's preferences might have consequences for a subject's own value computations. The presence of such plasticity would also be expected to induce behavioral change in the subject's own temporal discount rate, a parameter usually assumed to index a stable personality trait (Kirby, 2009; Ohmura et al., 2006). One can conjecture that such plasticity might underlie social conformity effects, where individuals adjust their beliefs or preferences to align more with those with whom they interact (Campbell-Meiklejohn et al., 2010; Edelson et al., 2011; Zaki et al., 2011).

At a neuronal level, a formal test of these predictions requires a fine-grained access to neural populations supporting distinct value computations, as well as a robust measure of learning-induced change in activity of these same populations. Despite its coarse spatial resolution, fMRI can reveal relationships

between underlying cellular representations. In particular, fMRI adaptation paradigms can be finessed to measure plastic changes associated with the behavioral pairing of different items (Barron et al., 2013; Klein-Flügge et al., 2013). The principle of fMRI adaptation builds on the idea that the repeated engagement of the same neuronal population leads to a diminished response and attenuated BOLD signal, even though the underlying biophysical mechanism remains ambiguous (Grill-Spector et al., 2006; Kohn, 2007).

Here we used an fMRI adaptation paradigm to measure the relationship between neuronal value representations for self, a familiar other whose preferences had been learnt prior to scanning and a novel confederate as this latter agent's preferences were learnt. We deployed a dynamic repetition suppression procedure to provide us with a probe of plastic neural changes associated with learning a new flexible computation. We hypothesized that plasticity associated with this new learning would impact upon the preference representation for self as a consequence of a neuronal representation that maps agent and offer onto an agent-independent measure of subjective value. In essence, this predicts that neuronal value representations between self and a novel other should become more similar with learning, in line with a behavioral shift in preference. An alternative hypothesis posits separate value computations for distinct agents. In such a case, a subject might use their own separate neural representations as a proxy for understanding another's traits, and an independent neuronal value representation for this other would be constructed through learning-induced plasticity (Barron et al., 2013). This alternative scenario predicts that neural value representations for self and other should become less similar with learning. In terms of a mechanism driving such plasticity, we reasoned that the same prediction errors that drive learning about a new partner's inter-temporal preferences would also induce shifts in the subject's own discount rate toward that of the partner.

RESULTS

Discount Rates Are Susceptible to Social Influence

To examine whether learning about the preferences of another agent impacts on subjective inter-temporal preferences, we tested 27 subjects on a standard inter-temporal choice task both before, and after, performing the identical task on behalf of a partner (Figures 1A and 1B). As in the standard format, subjects deciding for themselves chose between an immediately available smaller reward and a delayed larger reward. The degree to which delay diminishes the value of a reward was then quantified by a discount rate, computed from each subject's actual choices both before and after the experimental manipulation. The latter involved a context whereby subjects performed the very same task but now chose the option they inferred a confederate would prefer. After each trial they were given feedback about the choice the confederate had actually made, such that they could learn to simulate these choices in future trials.

Subjects learnt quickly, and accurately, to choose according to a novel partner's preferences (Figures S1C and S1D). Subjects believed that the partner was a human participant playing the game in a neighboring room (Figures S1G and S1H). In actual fact, and in part motivated by a need for good experimental control, we delivered feedback of a simulated player with preferences very different from the subjects' own (see Experimental Procedures).

Notably, we found that, after learning a partner's preferences, subjects' own discount rate shifted in the direction of the partner $((\log k_{self,block\ 3} - \log k_{self,block\ 1})/(\log k_{other,block\ 2} - \log k_{self,block\ 1})$, $t_{21} = 3.06$, $p = 0.006$, Figure 1C). Their estimate of the novel other's preferences remained stationary $((\log k_{other,block\ 3} - \log k_{other,block\ 2})/(\log k_{self,block\ 1} - \log k_{other,block\ 2})$, $t_{21} = 0.99$, $p = 0.33$) and was not biased toward subjects' own preferences ($t_{21} = 0.49$, $p = 0.63$). This effect is not easily understood as a social norm effect (Ruff et al., 2013), as we also observed discount rates shifted similarly when subjects were instructed they were deciding on behalf of a computer agent ($t_{22} = 3.89$, $p < 0.001$, Figure S1F).

One account of this shift in preference is that it arises out of a simulation of the other's preferences. In order to test whether such simulation is crucial for this shift or whether the behavior can be explained by simple stimulus- or action-based reinforcement, we designed a category-learning control experiment (Ashby and Maddox, 2005). This consisted of the same stimuli and actions, but the necessity to simulate another's discount rate was removed. Subjects were presented with a geometric depiction of a given choice on the screen (x axis: delay of the latter option; y axis: ratio of magnitudes M_{LL}/M_{SS}; Figure S1A, right) and instructed to choose according to the location of the dot with respect to an imaginary isoprobability line. Rather than using feedback to update a value simulation, subjects now updated their belief about the orientation of this line. In this scenario, subjects' discount rates did not shift, indicating that subjects were not merely repeating previous choices they had made on behalf of the other ($t_{24} = 0.61$, $p = 0.55$; see Figure S1F). This latter finding emphasizes a necessity for preference simulation for another agent in order to modulate a discount rate.

Subjective Value Changes Are Induced by Learning

The above account suggests that learning to compute the preferences of another agent induces plastic changes in the neural architecture responsible for personal valuation. This in turn predicts the neural population engaged during the computation of self valuation should change over the course of the experiment. This population should either become closer to that evoked during valuation for the partner if the representational structure of an offer depends solely on its subjective value irrespective of the individual. Alternatively, it should become less close if separate agent-specific representations exist and subjects construct an independent representation for the novel other as a consequence of learning. To test for such change in similarity between neural representations for self and others we interleaved trials from the delegated inter-temporal choice task with "probe" trials in the fMRI scanner. These probe trials enabled us to measure repetition suppression between individuals (Figures 1D and 1E). We reasoned that if self and partner valuation mechanisms overlapped more after learning than before, in line with an increase in behavioral similarity, then this predicts greater repetition suppression at the end of the experiment than at the beginning. If, however, subjects constructed a representation of the

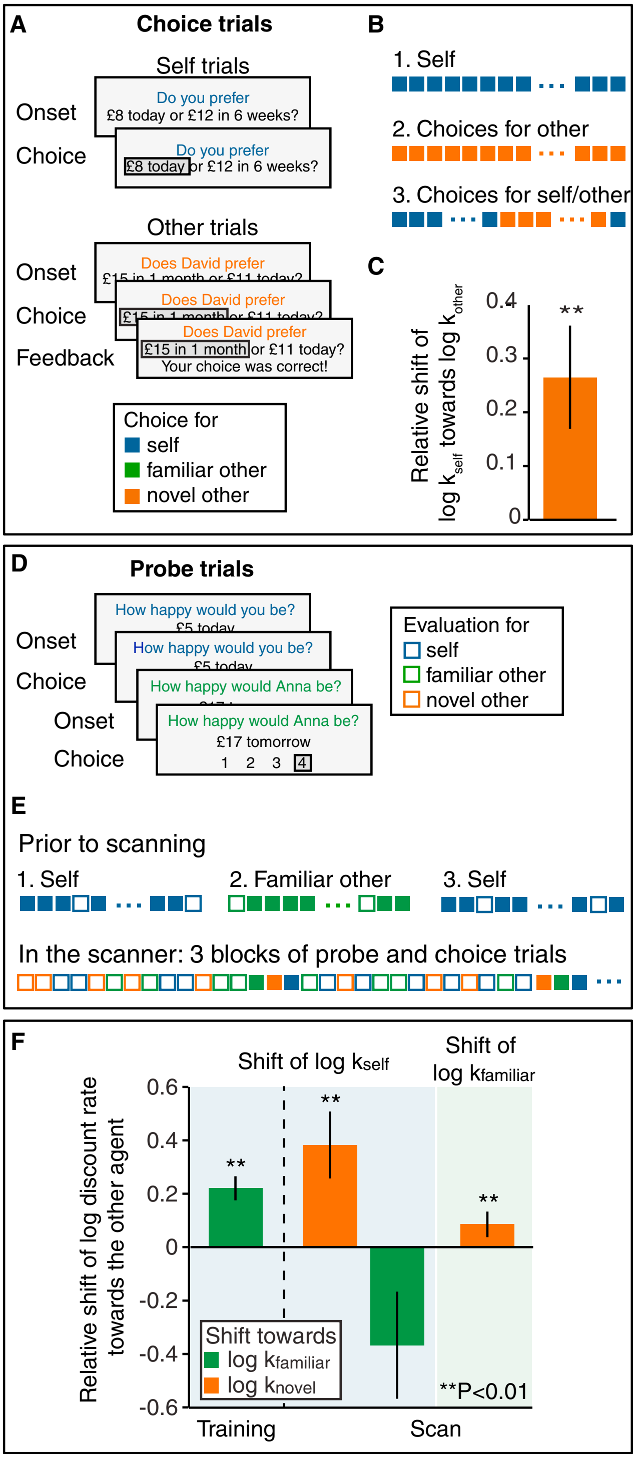

Figure 1. Experimental Design and Behavior

(A) On choice trials, subjects chose between an immediately available, smaller, and a delayed, larger reward. On "self" trials, subjects considered the choice for themselves. On "other" trials, they made the choice on behalf of a partner, and feedback indicated whether their choice corresponded to the partner's (simulated) choice.

(B) Structure of the behavioral experiment. Block 1 consisted of self choice trials alone, block 2 consisted of other choice trials alone, and block 3 consisted of alternating short blocks of 10 choice trials per agent (self or other).

(C) Shift of subjects' own discount rate (block 3 − block 1) relative to the distance between the estimated discount rate of the partner (block 2) and the initial discount rate for self (block 1), shift $= (log(k_{self,block\ 3}) - log(k_{self,block1}))/(log(k_{other,block\ 2}) - log(k_{self,block1}))$.

(D) The scanning version of the experiment also contained probe trials where subjects indicated on a four-item scale how happy an agent would be with the presented option.

(E) Prior to scanning, subjects' own discount rate was assessed before and after they were trained on the familiar other's preferences. In the scanner, subjects chose and evaluated for themselves, for the familiar other and for a novel other. The experiment was divided into three experimental blocks with probe trials the predominant type in all blocks.

(F) Relative shift of subjects' own discount rate (blue background) and the discount rate of the familiar other (green background) toward the familiar other (green bars) and the novel other (orange bars) during training and scanning. Data are represented as mean ± SEM. See also Figure S1.

novel other from their representation of self, then this predicts the very opposite, namely repetition suppression at the beginning of the experiment, which disappears as subjects build a separate representation of the novel partner.

To be certain that any effects were driven by learning about the partner, as opposed to exercising a choice per se, we introduced a third player (a familiar partner) whose discount rate had been learnt prior to scanning. This controlled for non-specific time-dependent signal changes not associated with learning of new preferences. Thus, our experiment comprised three players: the subject ("self"), a partner whose preferences were learnt prior to scanning ("familiar other"), and a partner whose preferences were learnt during scanning ("novel other"). The familiar and novel others' choices were simulated based on discount rates placed equally far apart on opposite, and counterbalanced, sides of the subject's original discount rate. This meant that one partner had a smaller, and the other partner a larger, discount rate than the subject himself.

We scanned 27 subjects while they performed the two interleaved tasks. In choice trials, as in the behavioral experiment described above, subjects again made inter-temporal choices for themselves and for the two partners. In "probe trials," subjects performed evaluations serially on behalf of different players, allowing us to measure repetition suppression between the value representations of different individuals (Figure 1E). After each choice trial for the novel or the familiar partner, but not after probe trials, subjects were given feedback about the choice the confederate had made.

In line with our behavioral results, subjects' discount rates shifted toward the discount rate of the familiar partner during preference learning prior to scanning (t_{23} = 3.17, p = 0.004, Figure 1F). During scanning, both subjects' own discount rate (t_{23} = 3.05, p = 0.006) and subjects' estimated discount rate of the familiar partner (t_{24} = 2.87, p = 0.008) shifted toward the newly learnt discount rate of the novel partner, with a stronger relative shift evident for subjects' own discount rate (t_{22} = 2.18, p = 0.04) but comparable absolute shifts (t_{22} = 0.72, p = 0.48). These preference shifts were therefore not simply associated with repeating the partner's choices but instead are most parsimoniously explained as induced by learning a new individual's preferences.

CellPress

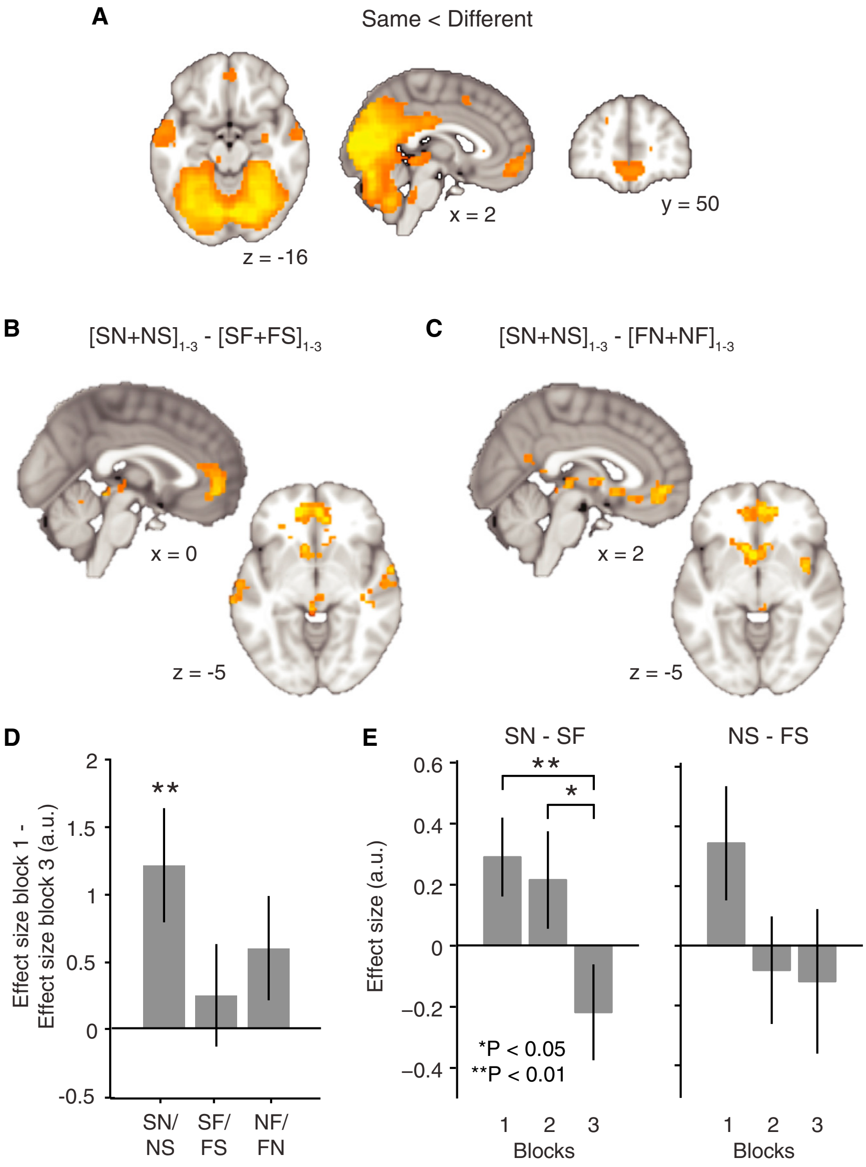

Figure 2. Learning-Induced Plasticity in mPFC

(A) Repetition suppression as an index of representational similarity. Displayed are brain areas with significantly less activity for repeated compared to changing agents on subsequent trials.

(B) Brain areas with a significantly greater increase in suppression from block 1 to block 3 between self and novel other compared to the increase in suppression between self and familiar other. This region overlaps with an area involved in self-referential processing and value coding (Figure S3).

(C) Areas displaying an increase in suppression from block 1 to block 3 between self and novel other relative to changes in suppression between novel and familiar other.

(D) Parameter estimates extracted by a jack-knife procedure from the mPFC ROI in Figure 2B, averaged across subjects. Visual areas do not show these selective suppression effects (Figure S2), and the neural suppression is not reflected in response times (Figure S4).

(E) Same parameter estimates as in (D) but now separated into the distinct components.

Data are represented as mean ± SEM. Contrast images in (A)–(C) are thresholded at $p < 0.01$ uncorrected for visualization. SN: novel-preceded-by-self; NS: self-preceded-by-novel; SF: familiar-preceded-by-self; FS: self-preceded-by-familiar; NF: familiar-preceded-by-novel; FN: novel-preceded-by-familiar. a.u.: arbitrary units.

Plasticity between Neural Representations of Self and Other

To address whether a measured change in subjective preference is linked to plasticity in neural populations computing valuations for self, we focused our analysis on the probe trials. We first established that we could measure repetition suppression by comparing brain activity elicited by simulating values for an agent when preceded by the same agent compared to a situation where an agent was preceded by another agent. Different agents were indicated to the subject by different colors on screen (Figure 1D). Unsurprisingly, we observed fMRI adaptation in the visual cortex ($p < 0.001$, peak $t_{26} = 16.93$, [30, −61, −8], reported here and in subsequent fMRI analyses as familywise error (FWE) corrected on cluster level, Figure 2A) (Buckner et al., 1998; Wiggs and Martin, 1998), but also in a network that included mPFC ($p = 0.02$, peak $t_{26} = 5.76$, [3, 53, −11]) and left superior temporal sulcus (STS) ($p < 0.001$, peak $t_{26} = 4.95$, [−51, −13, −8]). The latter two regions are associated with mentalizing (Gallagher and Frith, 2003), valuation for self (Boorman et al., 2009; Hunt et al., 2012; Kable and Glimcher, 2007), and valuation for others (Jenkins et al., 2008; Nicolle et al., 2012). While this main effect of repetition suppression does not dissociate visual from agent-specific effects, it confirms that similarity in neural patterns evoked in a valuation network can be indexed by repetition suppression (Barron et al., 2013; Jenkins et al., 2008).

We reasoned that we could use this index of neural similarity to investigate whether the observed shift in subjective preferences was linked to plastic changes in the valuation network. If the neural code depends on the subjective values of a given offer alone, then repetition suppression should emerge between self and novel other over the course of the experiment, given that discount rates for self align with discount rates for the novel other. If, on the other hand, the mPFC encodes value differentially depending on agent, where learning another's preferences involves the construction of an independent representation of this novel other from a representation of self, then repetition suppression should decrease over the course of the experiment. While a similar change in suppression might also be predicted between

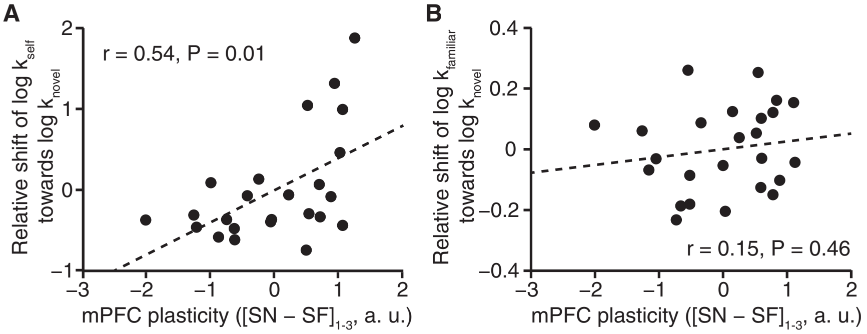

Figure 3. Relationship between $[SN - SF]_{1-3}$ Plasticity and Shift in Discount Rate

(A) Partial correlation between the change in suppression between self and novel relative to the change in suppression between self and familiar agents over blocks and the shift in subjects' own discount rate toward the novel other.

(B) Partial correlation between the change in suppression between self and novel relative to the change in suppression between self and familiar agents over blocks and the shift in subjects' estimate of the familiar other's discount rate toward the novel other.

Parameter estimates in (A) and (B) were extracted from the mPFC ROI shown in Figure 2B. To account for the correlation between subjects' own shift in discount rate and the shift in their estimate of the familiar other's discount rate, we performed partial correlations (i.e., the familiar shift was removed from behavior and neural signal in [A] and the self shift was removed from behavior and neural signal in [B]). The relationship between $[FN\text{-}SN]_{1\text{-}3}$ plasticity and the shift of the familiar other's discount rate toward the novel other is analyzed in Figure S5. a.u.: arbitrary units.

novel and familiar others, there should be no such change in suppression between self and familiar other if in fact we are indexing changes induced by new learning.

We designed a contrast that measured the change in repetition suppression between self and novel other from block 1 to block 3, controlled for by the change in repetition suppression between self and familiar other over the same blocks (see Experimental Procedures). The only brain region to survive whole-brain statistical correction was in mPFC (Figure 2B, $p = 0.01$, peak $t_{26} = 3.82$, [−12, 53, 1]), although sub-threshold clusters in the left and right STS were also present ($p = 0.27$, peak $t_{26} = 3.77$ and $p = 0.48$, peak $t_{26} = 3.38$, respectively). This region overlaps with an area involved in self-referential processing and in encoding value on probe trials (Figures S3B and S3C). There were no significant effects for the opposite interaction. This change cannot be due to visual effects, as we controlled for these both by inclusion of the familiar agent and separately by the comparison between early and late blocks in the experiment. Consequently, visual regions do not show these condition-specific changes in suppression (Figure S2). Neither can the effect be due to novelty or differences in processing speed, as no differences between main effects of novel and familiar others were seen in this region (Figure S3A) or in the response times (Figures S4A and S4B). Furthermore, an equivalent contrast measuring the change in suppression between self and novel other, but now controlling for the change in suppression between familiar and novel other, revealed a similar change in activity in an overlapping brain region (Figure 2C). Hence, in the mPFC learning the preferences of a novel agent specifically increased repetition suppression between representations of self and this novel partner.

To further investigate these mPFC suppression effects, we employed a jack-knife procedure across subjects to extract parameter estimates from the cluster of interest. Consistent with the whole-brain analysis, we found a significant change in novel-to-self/self-to-novel suppression (Figure 2D, $t_{26} = 2.86$, $p = 0.008$), but not in self-to-familiar/familiar-to-self suppression from block 1 to block 3 ($t_{26} = 0.64$, $p = 0.52$). The change in novel-to-familiar/familiar-to-novel suppression in the same region of interest (ROI) was in the same direction, but did not reach significance ($t_{26} = 1.54$, $p = 0.14$), and was smaller than the change in novel-to-self/self-to-novel suppression ($t_{26} = 1.65$, $p = 0.05$). Since overall activity in mPFC for self trials was greater than activity for other trials (Figure S3B), sensitivity to repetition suppression may differ depending on the order of the two agents. To explore potential differences, we decomposed the contrasts described above. Changes in repetition suppression between self and novel other were observed in both directions (Figure 2E) but were only significant when self trials were the priming and not the test trials (Figure 2E; ANOVA: left, $F_{2,78} = 3.39$, $p = 0.04$, right $F_{2,78} = 1.55$, $p = 0.21$).

Plasticity in mPFC Predicts Discount Rate Shifts

If the observed behavioral change in preference is related to learning-induced plasticity in value computations, then the increase in representational similarity between self and novel other should predict a subject's shift in preference. The increase in self-to-novel relative to self-to-familiar suppression over blocks did indeed predict the shift in subjects' own discount rate toward the novel other (partial correlation, $r = 0.54$, $p = 0.007$, Figure 3A), but not the same shift in the subjects' estimate of the familiar other's discount rate (partial correlation, $r = 0.15$, $p = 0.46$, Figure 3B), although a direct comparison of these effects in a multiple regression analysis did not reach significance ($t_{23} = 0.71$, $p = 0.24$). The shift in subjects' estimate of the familiar other's preferences was instead loosely related to an increase in representational similarity between familiar and novel other (Figure S5). The fact that the behavioral estimate for a shift in discount rate was derived from choice trials, whereas the neural plasticity effect was extracted from probe trials, strongly suggests that learning a partner's choice induces a stable plasticity in regions involved in value computation.

Plasticity in mPFC Is Predicted by Surprise Coding in the Striatum

A plausible mechanism for inducing plastic change is surprise or prediction error, which in this context arises when the familiar or the novel partner's choices diverge from the choice the subjects themselves would have made given the same choice context. Bayes-optimal estimates of this measure (see Experimental Procedures) were reflected in the posterior medial frontal cortex (pMFC) (Figure 4A, $p = 0.04$, peak $t_{26} = 4.09$, [−9, 29, 58]), a region previously associated with surprise coding in monkeys

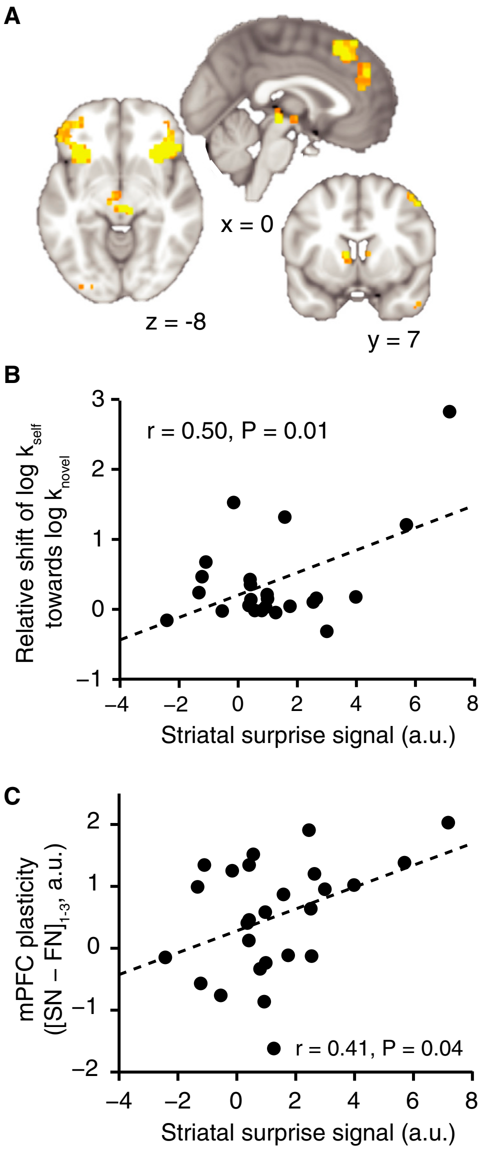

Figure 4. Surprise as a Mechanism Underlying mPFC Plasticity

(A) Brain areas correlating with the surprise subjects experienced when observing the novel and the familiar partners' choices.

(B) Correlation between the striatal correlate of the surprise about the novel other's choices, extracted from ROI in (A), and the shift of subjects' discount rates toward the novel other.

(C) Correlation between the striatal correlate of the surprise about the novel other's choices and $[SN - SF]_{1-3}$ plasticity in mPFC.

(A) is thresholded at $p < 0.01$ uncorrected for visualization. a.u.: arbitrary units.

(Hayden et al., 2011), as well as in both insula and striatum (caudate nucleus), although these did not survive a stringent multiple comparisons correction (right insula: p = 0.16, peak t_{26} = 8.37, [30, 26, −8]; left insula: p = 0.19, peak t_{26} = 6.25, [−33, 26, −5]; left striatum (p = 0.84, peak t_{26} = 3.44, [3, −25, −8]). pMFC and striatum are strongly implicated in the expression of a prediction error type signal in reinforcement learning (Pessiglione et al., 2006; Voon et al., 2010), as well as in signaling a discrepancy between an individual's behavior and the behavior of a group (Tomlin et al., 2013). An alternative measure of prediction error, where surprise was quantified as the discrepancy between the predicted choices of the partner and the partner's actual choices, did not yield significant activity in any area of the brain. A more lenient cluster-defining threshold of p = 0.05 revealed much smaller clusters in a similar network as the first surprise measure that did not survive multiple comparisons correction (e.g. pMFC, p = 1.0, peak t_{26} = 2.72, [6, 35, 40]).

A striatal prediction error type signal is known to drive learning through an influence on cortico-striatal plasticity (Reynolds and Wickens, 2002). In line with this notion, the BOLD correlate of the surprise about the novel partner's choices in the striatum predicted the behavioral shift in subjects' own discount rate (Figure 4B, r = 0.50, p = 0.01) as well as the change in self-to-novel versus change in self-to-familiar neuronal suppression over blocks in mPFC (Figure 4C, r = 0.41, p = 0.04). No such relationship was evident for pMFC or insula activity and mPFC plasticity (r = 0.04, p = 0.84 and r = 0.14, p = 0.48, respectively).

Finally, if prediction errors cause plasticity, and plasticity in turn causes the shift in subjects' discount rate, then plasticity in mPFC should formally mediate the impact of the striatal surprise signal on the shift in discount rate. We used single-level mediation to test this hypothesis (Wager et al., 2008). The path model jointly tests three effects required if indeed mPFC plasticity provides the link between a surprise signal and the shift in discount rate: namely, the relationship between striatal surprise effects and mPFC plasticity (path a), the relationship between mPFC plasticity and shift in discount rate (path b), and a formal mediation effect (path ab) that indicates that each explains a part of the discount rate shift covariance while controlling for effects attributable to the other mediator. All three effects were significant in a mediation analysis (path a = 0.15, SE = 0.07, p = 0.04; path b = 0.30, SE = 0.12, p < 0.001; path ab = 0.05, SE = 0.03, p = 0.01, Figure 5), supporting the idea that prediction errors influence the discount rate by inducing mPFC plasticity, which in turn impacts upon choice behavior. Hence, subjects with the largest striatal surprise signal at outcome of choice trials exhibited both the largest changes in representational similarity on probe trials and the largest changes in preferences, suggesting a role for striatal prediction error signals in inducing cortical plasticity and associated behavioral change.

DISCUSSION

The brain's representational architecture involves population codes wherein individual neurons contribute to a multitude of computations. We set out to investigate whether multiple neuronal representations can be updated simultaneously by learning-induced plasticity targeting one computation alone. The approach we developed exploited repetition suppression (Grill-Spector et al., 1999; Henson et al., 2000) to probe the similarity between distinct neural representations (Barron et al., 2013) by interleaving probe valuation trials with decision blocks that induced prediction errors and learning. While the biophysical mechanisms underlying fMRI repetition suppression remain ambiguous (Sobotka and Ringo, 1994), in a careful experimental design this approach allows inferences about population coding

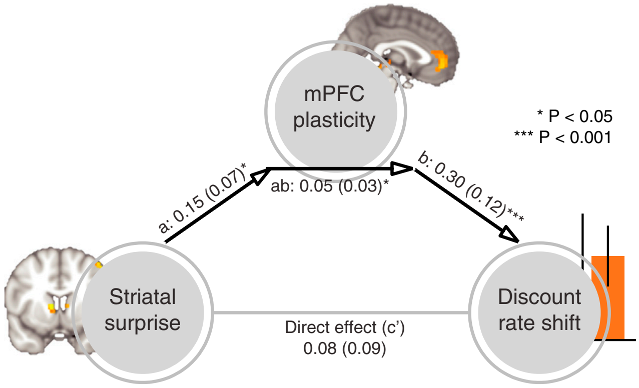

Figure 5. Mediation Path Diagram for Discount Rate Shift as Predicted from a Striatal Surprise Signal

The striatal correlate of the surprise about the novel other's choices predicted $[SN - SF]_{1-3}$ plasticity in the medial prefrontal cortex (path a), and the mediator (mPFC plasticity) predicted the shift of subjects' own discount rate toward the discount rate of the novel other (path b, controlled for the striatal surprise signal). Importantly, there was a significant mediation effect (path ab), indicating that mPFC plasticity formally mediates the relationship between striatal surprise and the shift in discount rate. The direct path between striatal surprise and shift in discount rate, controlled for both mediators, was not significant (path c'). The lines are labeled with path coefficients (SEs).

with respect to precise features of stimuli (Kourtzi and Kanwisher, 2001) or computations (Barron et al., 2013; Doeller et al., 2010).

We were interested in changes of value representational similarity over time. By asking subjects to evaluate presented options on behalf of themselves, a novel other whose preferences were acquired during on-line scanning and a familiar other whose preferences had previously been learnt, we could interrogate representational similarity in neuronal populations encoding valuation for these three agents. In line with previous reports that highlight a social influence on the valuation of objects (Campbell-Meiklejohn et al., 2010; Klucharev et al., 2009; Zaki et al., 2011), we found learning about the preferences of a novel agent had clear behavioral consequences evident in a shift in subjects' own, as well as their estimation of a familiar other's, discount rate. This behavioral effect coincided with an increase in neural representational similarity in the mPFC. This supports a view that value representations in the mPFC are not aligned to the frame of reference of an individual. Instead, the increase in neuronal overlap tied to a shift in behavioral preferences suggests that the mPFC encodes agent-independent representations of subjective value.

The presence of a learning-induced representational plasticity for value is likely to depend on generic learning mechanisms. The most influential computational account posits a role for a reward prediction error implemented via phasic activity of dopamine neurons (Schultz et al., 1997), a putative teaching signal for cortico-striatal learning (Calabresi et al., 2007; O'Doherty et al., 2004; Reynolds and Wickens, 2002). Prediction errors align with the dimension relevant for learning in a given situation. They manifest as a sensory prediction error when subjects learn to predict a sensory event (den Ouden et al., 2010), a probability prediction error when subjects learn about reward probability (Behrens et al., 2008), and a social expectancy prediction error when group preferences diverge from subjects' own valuations (Campbell-Meiklejohn et al., 2010; Klucharev et al., 2009). In the current experiment, a prediction error (expressed in pMFC, insula, and striatum) corresponds to the surprise subjects experience when a partner's choice is incongruent with their own preference. This accords with previous studies demonstrating an expression of a similar signal representing a discrepancy between one's own and a group's opinion (Berns et al., 2010; Campbell-Meiklejohn et al., 2010; Falk et al., 2010; Klucharev et al., 2009). Crucially, our results extend on these reports by showing this error coding is directly related to an expression of plasticity in mPFC, a region widely implicated in tracking preferences for stimuli (Lebreton et al., 2009) as well as inter-temporal preferences (Kable and Glimcher, 2007; Pine et al., 2009).

The mPFC region displaying the change in repetition suppression is a complex and heterogeneous area with strong connections to regions such as the amygdala, hippocampus, hypothalamus, and insula enabling access to sensory, visceral, and emotional information. It is considered ideally placed for self-referential processing (Kelley et al., 2002; Magno and Allan, 2007) and for attributing value to stimuli across many reward contexts (Bartra et al., 2013; Clithero and Rangel, 2014) and internally generated states (Bouret and Richmond, 2010). However, a mPFC value computation is also remarkably flexible, and can occur even if direct experience is not available (Barron et al., 2013) or if there is a requirement for an abstract model of task structure (Hampton et al., 2006). This flexibility is vital in social cognition, where a model of the preferences and intentions of another individual needs to be decoupled from the physical and perceptual reality of a subject's own internal state (Mitchell, 2009; Nicolle et al., 2012). Traditionally, it has been suggested that such computations occur in distinct circuitries, where a ventral sector of the mPFC encoding subjective stimulus values (Boorman et al., 2009; O'Doherty, 2004) is complemented by a dorsal sector representing the mental states of others (Behrens et al., 2008, 2009; Frith and Frith, 2010; Yoshida et al., 2010). However, this notion is challenged by an observation that a dorsal-ventral axis can be better understood in terms of executed versus modeled choices (Nicolle et al., 2012). The latter observation supports the idea that the very same area encodes subjective value irrespective of the frame of reference, a notion strongly supported by our current observation that a behavioral shift toward the value of a novel agent is mirrored by an increase in neural overlap.

Irrespective of the exact nature of the observed plasticity, the underlying mechanism would seem to necessitate an overlap in neural populations encoding values for a novel other, self, and a familiar other. How exactly might the brain calculate discounting preferences with neural populations that are prone to the observed shifts in preference? Theoretical studies suggest an agent's overall preferences might arise out of a summation over a distributed set of discounting units (Kurth-Nelson and Redish, 2009). This is consistent with recordings in rat orbitofrontal cortex demonstrating a distributed encoding of inter-temporal choice parameters across a neuronal population (Roesch et al., 2006). Similar gradients of discount factors have also

been found in the human striatum (Tanaka et al., 2004) and mPFC (Wang et al., 2014). This suggests that some neuronal assemblies may represent a preference for fast discounting, favoring smaller-sooner returns, while others favor slow discounting. The discounting preference of each agent would be represented by population codes, implementing sets of weights over these discounting assemblies. The prediction errors a subject perceives when the novel other's choices differ from what they would have chosen for themselves could in principle change the weights within this pool, resulting in altered populations codes.

The fact that a common brain region is recruited when computing preferences for self and other might suggest that people initially draw on self-representations to make inferences about another person and only construct a novel representation through learning. Such a mechanism has been observed when constructing a representation for a novel good from a simultaneous activation of familiar components (Barron et al., 2013). However, this theory makes opposite neural predictions, as it predicts repetition suppression at the beginning of the experiment as subjects draw on the same representation to choose for self and other. In this scenario a separate representation for a novel other is built over time and would predict disappearance of repetition suppression. Instead, we observe an increase in repetition suppression across time, an effect reminiscent of an increase in similarity between representations observed when subjects repeatedly evoke independent memories (Barron et al., 2013). Importantly, we can demonstrate this plasticity is not solely a neuronal phenomenon but also has profound behavioral consequences.

Our approach uses repetition suppression to provide insight into a similarity in neural representations. Comparable measures of representational content can be obtained by multivariate pattern analysis (Davis and Poldrack, 2013; Sapountzis et al., 2010); however, it is thought the two techniques show a difference in sensitivity to precise features of the neuronal code (Drucker and Aguirre, 2009). Without an explicit measure of MVPA in this study, we are therefore cautious in predicting a comparable increase in similarity between representations for self and a novel other in mPFC when using MVPA.

Note that subjects grow increasingly familiar with the novel other's preferences as the task progresses, whereas familiarity remains constant for the familiar other in the sense that there is no new learning in relation to this other. Since psychological constructs such as familiarity, but also similarity and physical proximity, have previously been demonstrated to upregulate mPFC activity (Jenkins et al., 2008; Krienen et al., 2010; Mitchell et al., 2006; Tamir and Mitchell, 2011), this raises the question whether an increase in familiarity might drive the plasticity effect. Importantly, our data are not consistent with such an account. First, activity for familiar and novel other does not differ in mPFC, not even at the beginning of the experiment, suggesting that the mPFC in our task does not respond to familiarity per se. Second, a mediation analysis suggests that it is a striatal surprise signal, the very opposite of familiarity, that drives the plasticity effect, which in turn drives the behavioral shift.

Subjects' own discount rate shifted toward the discount rate of their partner irrespective of whether their partner was human or a computer. This is in line with studies demonstrating that individuals use strategies akin to those used in real social contexts when interacting with a computer agent (Nass and Moon, 2000). Crucially, a control condition with the same stimuli and actions, but without the need to employ a discounting computation, did not evoke a change in subjects' own preferences. This indicates that the behavioral effect is tied to subjects' deployment of the very same discounting mechanism to learn on behalf of another agent, be it a human or non-human agent. Thus, it is a learning-induced plasticity in acquiring a novel value representation that impacted on subjects' own subjective value computation. This also suggests that most subjects do not actively choose to change their preferences but instead do so as the consequence of an mPFC plasticity they are not consciously aware of. Such an implicit mechanism presumably contributes to involuntarily aligning goals with others and might play an important role in spreading values throughout a population (Boyd et al., 2011; Frith and Frith, 2010).

In conclusion, our data detail a neuronal mechanism by which personal traits are susceptible to social influence. Such plasticity might be one of the key features underlying learning, because it allows for an integration of past experience with novel information. More broadly, our findings pave the way for further studies of human social interactions at a more mechanistic level.

EXPERIMENTAL PROCEDURES

Subjects

27 volunteers (mean age ± SD: 23.6 ± 3.7, 14 females) participated in the behavioral experiment, and 29 volunteers (mean age ± SD: 25.6 ± 5.6 years, 14 females) participated in the subsequent fMRI experiment. Two subjects were excluded from fMRI analyses, because they had previously participated in the behavioral experiment and because of technical difficulties during the scan. All subjects were neurologically and psychiatrically healthy. The study took place at the Wellcome Trust Centre for Neuroimaging in London, UK. The experimental procedure was approved by the University College London Hospitals Ethics Committee and written informed consent was obtained from all subjects.

Task Behavioral Study

For a detailed description of the task and our analyses, see the Supplemental Information. In brief, subjects made a series of choices between a smaller amount paid on the same day and a larger amount paid later (Figure 1A). The experiment was divided into three blocks (Figure 1B). In the first block, consisting of 100 trials, subjects made decisions for themselves. In block 2, they made decisions on behalf of their partner. They were also provided with trial-by-trial feedback on whether their choice for the partner was correct. Block 2 ended when subjects made 85% correct responses for their partner in a sliding window of 20 trials or after a maximum of 60 trials. In block 3, smaller blocks of ten trials of choosing for self alternated with blocks of ten trials of choosing for the partner. Block 3 ended after a total of 200 trials. Choices were optimized to give us a precise estimate of subjects' discount rates.

Estimation of Discount Rates

We estimated subjects' discount rates by fitting a hyperbolic model to their choices (Rachlin et al., 1991) separately for each experimental block. Subjects' shift in discount rates was defined as the change in discount rate from block 1 to block 3 ($\log k_{self,block3} - \log k_{self,block1}$) relative to the distance between their estimate of the partner's discount rate from their own discount rate ($\log k_{other,block2} - \log k_{self,block1}$):

$$(1)\ shift = \frac{\log k_{self,block\ 3} - \log k_{self,block1}}{\log k_{other,block\ 2} - \log k_{self,block1}}.$$

A positive shift represents a movement toward, and a negative shift a movement away from, the partner's discount rate. Outliers (outside the range mean ± 3•SD), as well as subjects who estimated their partner's discount rate to be less than 0.3 units away from their own discount rate, were excluded from population analyses because of inflated shift estimates (see Figure S1E).

Simulation of the Other's Choices

To generate feedback for the confederate's choices, we simulated a partner with a discount rate that differed from the subject's own baseline discount rate by 1 (i.e., $\log k_{other} = \log k_{self,block1} \pm 1$). Choices were correct if they corresponded to the decision that would be preferred by a hyperbolic discounter with this discount rate. Importantly, the simulated partner's choices were noisy, as the other's subjective value was translated to a choice probability with a softmax function (temperature parameter $\beta = 1$).

Task fMRI Study

The fMRI experiment consisted of two trial types: choice trials, as described for the behavioral experiment above, and probe trials, in which subjects evaluated a single option on a scale from 1 to 4 (Figure 1D). Subjects learned the preferences of a second partner ("familiar other") before the scan (Figure 1E, top).

In contrast to the behavioral experiment and the pretraining, subjects learned about the novel other's discount rate while we assessed their own discount rate. To make sure that we captured a potential shift in discount rate in this scenario, we excluded the first third of all choice trials subjects performed in the scanner when estimating $k_{self,scan}$, $k_{novel,scan}$, and $k_{familiar,scan}$. The relative shift effects reported in Figure 1F were then calculated as follows:

$$(4)\ shift_{self\rightarrow fam,training} = \frac{\log k_{self,training\_block\ 3} - \log k_{self,training\_block1}}{\log k_{familiar,training\_block2} - \log k_{self,training\_block1}}$$

$$(5)\ shift_{self\rightarrow fam,scan} = \frac{\log k_{self,scan} - \log k_{self,training\_block3}}{\log k_{familiar,scan} - \log k_{self,training\_block3}}$$

$$(6)\ shift_{self\rightarrow novel,scan} = \frac{\log k_{self,scan} - \log k_{self,training\_block3}}{\log k_{novel,scan} - \log k_{self,training\_block3}}$$

$$(5)\ shift_{fam\rightarrow novel,scan} = \frac{\log k_{familiar,scan} - \log k_{familiar,training\_block2}}{\log k_{novel,scan} - \log k_{familiar,training\_block2}}.$$

For the estimation of absolute shifts, the denominator z was set to sign(z). Outliers (outside the range mean ± 3*SD) as well as subjects for whom the denominator was smaller than 0.3 (two subjects for $shift_{self\rightarrow fam,scan}$, three subjects for $shift_{self\rightarrow novel,scan}$, and two subjects for $shift_{fam\rightarrow novel,scan}$) were excluded from the analyses.

Surprise Measure

We estimated subjects' own discount rates on a trial-by-trial basis (see Supplemental Information) and used this measure to compute differences in subjective value for the choices subjects observed their partner make ($V_{chosen\_by\_partner} - V_{unchosen\_by\_partner}$). This difference in subjective value was transformed to a probability using a softmax function applied to a trial-to-trial estimation of subject's inverse temperature parameter β. This measure gave us an estimate of how likely the subject would have been to make the same choice himself. We subtracted this likelihood from 1 to translate this to a surprise measure.

Scan Procedure, fMRI Data Acquisition, and Preprocessing

We used standard procedures for acquiring fMRI data where these were designed to minimize susceptibility related artifacts in the ventral prefrontal cortex (Weiskopf et al., 2006). We used SPM8 for image preprocessing and data analysis (Wellcome Trust Centre for Neuroimaging, London). We corrected for signal bias, co-registered functional scans to the first volume in the sequence, and corrected for distortions using the fieldmap. Data were spatially normalized to a standard EPI template and smoothed using an 8 mm full-width at half maximum Gaussian kernel.

fMRI Data Analysis

Data were analyzed with an event-related general linear model (GLM). Probe trials were sorted into nine different conditions (self preceded by self [SS], novel preceded by self [SN], familiar preceded by self [SF], self preceded by novel [NS], novel preceded by novel [NN], familiar preceded by novel [NF], self preceded by familiar [FS], novel preceded by familiar [FN], and familiar preceded by familiar [FF]) with 20 trials per condition and block. Each regressor was accompanied by a parametric modulator reflecting subjective value from the respective agent's perspective. This value was calculated based on a trial-by-trial estimate of the subject's current belief about their partners' discount rate k. Furthermore, we defined one choice regressor per agent and block indexing the time at which subjects indicated their decision on choice trials and received feedback. Each was accompanied by a parametric regressor corresponding to the surprise subjects experienced as they observed the partner's choice. Button presses were included as a regressor of no interest. Because of the sensitivity of the BOLD signal in the OFC region to subject motion and physiological noise, we included six motion regressors obtained during realignment as well as ten regressors for cardiac phase, six for respiratory phase, and one for respiratory volume extracted with an in-house-developed Matlab toolbox as nuisance regressors (Hutton et al., 2011). Blocks were modeled separately within the GLM.

To detect areas showing adaptation to repeated agents as depicted in Figure 2A, we used the contrast $([\text{agent preceded by different agent}] - [\text{agent preceded by same agent}])$ (i.e., $([SN + SF + NS + NF + FS + FN] - 2[SS + FF + NN])$). To test for areas displaying greater increases in suppression between self and the novel other compared to between self and familiar other (Figure 2B), we defined the following contrast: $([SN + NS]_{block1} - [SN + NS]_{block3}) - ([SF + FS]_{block1} - [SF + FS]_{block3})$. To test for greater increases in suppression between self and novel other than between novel other and familiar other, we defined a contrast as follows: $([SN + NS]_{block1} + [SN + NS]_{block3}) - ([NF + FN]_{block1} - [NF + FN]_{block3})$.

The contrast images of all subjects from the first level were analyzed as a second-level random effects analysis. Results are reported at a cluster-defining threshold of $p < 0.01$ uncorrected combined with a FWE-corrected significance level of $p < 0.05$.

We performed a jack-knife procedure from the mPFC ROI (Figure 2B) to extract parameter estimates from this region without biasing the selection. To this end, we extracted parameter estimates for each subject from an ROI defined according to all other subjects (threshold at $p < 0.01$ uncorrected). This signal was used to perform all analyses depicted in Figures 2–4 and S3A.

We performed partial correlations to control for correlations between $shift_{self\rightarrow novel,scan}$ and $shift_{fam\rightarrow novel,scan}$ in our analysis of the relationship of a behavioral shift effect and neural plasticity. This removes the shift of the familiar other toward the novel other from the subjects' own discount rate shifts and the neural plasticity index $[SN - SF]_{1-3}$ (Figure 3A) and the shift of self toward the novel other from the familiar other's shift toward the novel other and the neural plasticity index (Figure 3B). We also estimated a linear regression model on the same data with $shift_{self\rightarrow novel,scan}$ and $shift_{fam\rightarrow novel,scan}$ as independent variables and $[SN - SF]_{1-3}$ as the dependent variable. The relationship between $shift_{self\rightarrow novel,scan}$ and $[SN - SF]_{1-3}$ was directly contrasted with the relationship between $shift_{fam\rightarrow novel,scan}$ and $[SN - SF]_{1-3}$.

To test for the influence of surprise on mPFC plasticity, we defined a contrast assessing BOLD correlate of the surprise subjects experienced as they got feedback about the novel and the familiar partners' choices. This contrast revealed activity in ACC, in bilateral insula and dorsal striatum (Figure 4A; note that insula and striatal activity did not survive cluster-based FEW thresholding). To identify the surprise experienced when learning about the novel other, parameter estimates were then extracted from these ROIs for the novel other's choices only. This surprise measure in the striatum was correlated with subjects' shift in discount rate (Figure 4B) and the plasticity measure $[SN - SF]_{1-3}$ extracted from the mPFC ROI (Figure 4C).

We used the Mediation and Moderation Toolbox (Atlas et al., 2010; Wager et al., 2008) to perform a mediation analysis on this surprise signal, our plasticity measure, and the discount rate shift.

To test the specificity of adaptation effects, we analyzed repetition suppression effects in visual regions. We defined an ROI from a contrast identifying a main effect to any visual event, averaged across all blocks, and performed the

same analyses as for the mPFC ROI (thresholded at $p < 0.0001$ uncorrected; Figure S2).

SUPPLEMENTAL INFORMATION

Supplemental Information includes five figures and Supplemental Experimental Procedures and can be found with this article online at http://dx.doi.org/10.1016/j.neuron.2014.12.033.

ACKNOWLEDGMENTS

We thank Helen Barron, Erie Boorman, Molly Crockett, Laurence Hunt, and Robb Rutledge for discussions and comments on an earlier draft of the manuscript. This work was supported by the Wellcome trust (4-year PhD studentship 097267/Z/11/Z to M.M.G.; Career Development Fellowship WT088312AIA and Senior Research Fellowship WT104765MA to T.E.J.B. and Senior Investigator Award 098362/Z/12/Z to R.J.D.), the Biomedical Research Council (M.M.), the Joint Initiative on Computational Psychiatry and Ageing Research between the Max Planck Society and University College London (Z.K.N.) and the James S. McDonnell Foundation (T.E.J.B.). The Wellcome Trust Centre for Neuroimaging is supported by core funding from the Wellcome Trust (Strategic Award Grant 091593/Z/10/Z).

Accepted: December 10, 2014
Published: January 21, 2015

REFERENCES

Ashby, F.G., and Maddox, W.T. (2005). Human category learning. Annu. Rev. Psychol. *56*, 149–178.

Atlas, L.Y., Bolger, N., Lindquist, M.A., and Wager, T.D. (2010). Brain mediators of predictive cue effects on perceived pain. J. Neurosci. *30*, 12964–12977.

Barron, H.C., Dolan, R.J., and Behrens, T.E.J. (2013). Online evaluation of novel choices by simultaneous representation of multiple memories. Nat. Neurosci. *16*, 1492–1498.

Bartra, O., McGuire, J.T., and Kable, J.W. (2013). The valuation system: a coordinate-based meta-analysis of BOLD fMRI experiments examining neural correlates of subjective value. Neuroimage *76*, 412–427.

Behrens, T.E.J., Hunt, L.T., Woolrich, M.W., and Rushworth, M.F.S. (2008). Associative learning of social value. Nature *456*, 245–249.

Behrens, T.E.J., Hunt, L.T., and Rushworth, M.F.S. (2009). The computation of social behavior. Science *324*, 1160–1164.

Berns, G.S., Capra, C.M., Moore, S., and Noussair, C. (2010). Neural mechanisms of the influence of popularity on adolescent ratings of music. Neuroimage *49*, 2687–2696.

Boorman, E.D., Behrens, T.E.J., Woolrich, M.W., and Rushworth, M.F.S. (2009). How green is the grass on the other side? Frontopolar cortex and the evidence in favor of alternative courses of action. Neuron *62*, 733–743.

Bouret, S., and Richmond, B.J. (2010). Ventromedial and orbital prefrontal neurons differentially encode internally and externally driven motivational values in monkeys. J. Neurosci. *30*, 8591–8601.

Boyd, R., Richerson, P.J., and Henrich, J. (2011). The cultural niche: why social learning is essential for human adaptation. Proc. Natl. Acad. Sci. USA *108* (*Suppl 2*), 10918–10925.

Buckner, R.L., Goodman, J., Burock, M., Rotte, M., Koutstaal, W., Schacter, D., Rosen, B., and Dale, A.M. (1998). Functional-anatomic correlates of object priming in humans revealed by rapid presentation event-related fMRI. Neuron *20*, 285–296.

Calabresi, P., Picconi, B., Tozzi, A., and Di Filippo, M. (2007). Dopamine-mediated regulation of corticostriatal synaptic plasticity. Trends Neurosci. *30*, 211–219.

Campbell-Meiklejohn, D.K., Bach, D.R., Roepstorff, A., Dolan, R.J., and Frith, C.D. (2010). How the opinion of others affects our valuation of objects. Curr. Biol. *20*, 1165–1170.

Clithero, J.A., and Rangel, A. (2014). Informatic parcellation of the network involved in the computation of subjective value. Soc. Cogn. Affect. Neurosci. *9*, 1289–1302.

D'Argembeau, A., Ruby, P., Collette, F., Degueldre, C., Balteau, E., Luxen, A., Maquet, P., and Salmon, E. (2007). Distinct regions of the medial prefrontal cortex are associated with self-referential processing and perspective taking. J. Cogn. Neurosci. *19*, 935–944.

Davis, T., and Poldrack, R.A. (2013). Measuring neural representations with fMRI: practices and pitfalls: Representational analysis using fMRI. Ann. N.Y. Acad. Sci. *1296*, 108–134.

den Ouden, H.E.M., Daunizeau, J., Roiser, J., Friston, K.J., and Stephan, K.E. (2010). Striatal prediction error modulates cortical coupling. J. Neurosci. *30*, 3210–3219.

Denny, B.T., Kober, H., Wager, T.D., and Ochsner, K.N. (2012). A meta-analysis of functional neuroimaging studies of self- and other judgments reveals a spatial gradient for mentalizing in medial prefrontal cortex. J. Cogn. Neurosci. *24*, 1742–1752.

Doeller, C.F., Barry, C., and Burgess, N. (2010). Evidence for grid cells in a human memory network. Nature *463*, 657–661.

Drucker, D.M., and Aguirre, G.K. (2009). Different spatial scales of shape similarity representation in lateral and ventral LOC. Cereb. Cortex *19*, 2269–2280.

Edelson, M., Sharot, T., Dolan, R.J., and Dudai, Y. (2011). Following the crowd: brain substrates of long-term memory conformity. Science *333*, 108–111.

Ersner-Hershfield, H., Wimmer, G.E., and Knutson, B. (2009). Saving for the future self: neural measures of future self-continuity predict temporal discounting. Soc. Cogn. Affect. Neurosci. *4*, 85–92.

Evenden, J.L. (1999). Varieties of impulsivity. Psychopharmacology (Berl.) *146*, 348–361.

Falk, E.B., Berkman, E.T., Mann, T., Harrison, B., and Lieberman, M.D. (2010). Predicting persuasion-induced behavior change from the brain. J. Neurosci. *30*, 8421–8424.

Frith, U., and Frith, C. (2010). The social brain: allowing humans to boldly go where no other species has been. Philos. Trans. R. Soc. Lond. B Biol. Sci. *365*, 165–176.

Gallagher, H.L., and Frith, C.D. (2003). Functional imaging of 'theory of mind'. Trends Cogn. Sci. *7*, 77–83.

Grill-Spector, K., Kushnir, T., Edelman, S., Avidan, G., Itzchak, Y., and Malach, R. (1999). Differential processing of objects under various viewing conditions in the human lateral occipital complex. Neuron *24*, 187–203.

Grill-Spector, K., Henson, R., and Martin, A. (2006). Repetition and the brain: neural models of stimulus-specific effects. Trends Cogn. Sci. *10*, 14–23.

Hampton, A.N., Bossaerts, P., and O'Doherty, J.P. (2006). The role of the ventromedial prefrontal cortex in abstract state-based inference during decision making in humans. J. Neurosci. *26*, 8360–8367.

Hayden, B.Y., Heilbronner, S.R., Pearson, J.M., and Platt, M.L. (2011). Surprise signals in anterior cingulate cortex: neuronal encoding of unsigned reward prediction errors driving adjustment in behavior. J. Neurosci. *31*, 4178–4187.

Henson, R., Shallice, T., and Dolan, R. (2000). Neuroimaging evidence for dissociable forms of repetition priming. Science *287*, 1269–1272.

Hunt, L.T., Kolling, N., Soltani, A., Woolrich, M.W., Rushworth, M.F.S., and Behrens, T.E.J. (2012). Mechanisms underlying cortical activity during value-guided choice. Nat. Neurosci. *15*, 470–476, S1–S3.

Hutton, C., Josephs, O., Stadler, J., Featherstone, E., Reid, A., Speck, O., Bernarding, J., and Weiskopf, N. (2011). The impact of physiological noise correction on fMRI at 7 T. Neuroimage *57*, 101–112.

Jenkins, A.C., Macrae, C.N., and Mitchell, J.P. (2008). Repetition suppression of ventromedial prefrontal activity during judgments of self and others. Proc. Natl. Acad. Sci. USA *105*, 4507–4512.

Kable, J.W., and Glimcher, P.W. (2007). The neural correlates of subjective value during intertemporal choice. Nat. Neurosci. *10*, 1625–1633.

Kelley, W.M., Macrae, C.N., Wyland, C.L., Caglar, S., Inati, S., and Heatherton, T.F. (2002). Finding the self? An event-related fMRI study. J. Cogn. Neurosci. *14*, 785–794.

Kirby, K.N. (2009). One-year temporal stability of delay-discount rates. Psychon. Bull. Rev. *16*, 457–462.

Klein-Flügge, M.C., Barron, H.C., Brodersen, K.H., Dolan, R.J., and Behrens, T.E.J. (2013). Segregated encoding of reward-identity and stimulus-reward associations in human orbitofrontal cortex. J. Neurosci. *33*, 3202–3211.

Klucharev, V., Hytönen, K., Rijpkema, M., Smidts, A., and Fernández, G. (2009). Reinforcement learning signal predicts social conformity. Neuron *61*, 140–151.

Kohn, A. (2007). Visual adaptation: physiology, mechanisms, and functional benefits. J. Neurophysiol. *97*, 3155–3164.

Kourtzi, Z., and Kanwisher, N. (2001). Representation of perceived object shape by the human lateral occipital complex. Science *293*, 1506–1509.

Krienen, F.M., Tu, P.-C., and Buckner, R.L. (2010). Clan mentality: evidence that the medial prefrontal cortex responds to close others. J. Neurosci. *30*, 13906–13915.

Kurth-Nelson, Z., and Redish, A.D. (2009). Temporal-difference reinforcement learning with distributed representations. PLoS ONE *4*, e7362.

Lebreton, M., Jorge, S., Michel, V., Thirion, B., and Pessiglione, M. (2009). An automatic valuation system in the human brain: evidence from functional neuroimaging. Neuron *64*, 431–439.

Magno, E., and Allan, K. (2007). Self-reference during explicit memory retrieval: an event-related potential analysis. Psychol. Sci. *18*, 672–677.

Mitchell, J.P. (2009). Inferences about mental states. Philos. Trans. R. Soc. Lond. B Biol. Sci. *364*, 1309–1316.

Mitchell, J.P., Macrae, C.N., and Banaji, M.R. (2006). Dissociable medial prefrontal contributions to judgments of similar and dissimilar others. Neuron *50*, 655–663.

Mitchell, J.P., Schirmer, J., Ames, D.L., and Gilbert, D.T. (2011). Medial prefrontal cortex predicts intertemporal choice. J. Cogn. Neurosci. *23*, 857–866.

Myerson, J., and Green, L. (1995). Discounting of delayed rewards: Models of individual choice. J. Exp. Anal. Behav. *64*, 263–276.

Nass, C., and Moon, Y. (2000). Machines and mindlessness: Social responses to computers. J. Soc. Issues *56*, 81–103.

Nicolle, A., Klein-Flügge, M.C., Hunt, L.T., Vlaev, I., Dolan, R.J., and Behrens, T.E.J. (2012). An agent independent axis for executed and modeled choice in medial prefrontal cortex. Neuron *75*, 1114–1121.

O'Doherty, J.P. (2004). Reward representations and reward-related learning in the human brain: insights from neuroimaging. Curr. Opin. Neurobiol. *14*, 769–776.

O'Doherty, J., Dayan, P., Schultz, J., Deichmann, R., Friston, K., and Dolan, R.J. (2004). Dissociable roles of ventral and dorsal striatum in instrumental conditioning. Science *304*, 452–454.

Ohmura, Y., Takahashi, T., Kitamura, N., and Wehr, P. (2006). Three-month stability of delay and probability discounting measures. Exp. Clin. Psychopharmacol. *14*, 318–328.

Pessiglione, M., Seymour, B., Flandin, G., Dolan, R.J., and Frith, C.D. (2006). Dopamine-dependent prediction errors underpin reward-seeking behaviour in humans. Nature *442*, 1042–1045.

Peters, J., and Büchel, C. (2010). Episodic future thinking reduces reward delay discounting through an enhancement of prefrontal-mediotemporal interactions. Neuron *66*, 138–148.

Pine, A., Seymour, B., Roiser, J.P., Bossaerts, P., Friston, K.J., Curran, H.V., and Dolan, R.J. (2009). Encoding of marginal utility across time in the human brain. J. Neurosci. *29*, 9575–9581.

Rachlin, H., Raineri, A., and Cross, D. (1991). Subjective probability and delay. J. Exp. Anal. Behav. *55*, 233–244.

Reynolds, J.N.J., and Wickens, J.R. (2002). Dopamine-dependent plasticity of corticostriatal synapses. Neural Netw. *15*, 507–521.

Robbins, T.W., Gillan, C.M., Smith, D.G., de Wit, S., and Ersche, K.D. (2012). Neurocognitive endophenotypes of impulsivity and compulsivity: towards dimensional psychiatry. Trends Cogn. Sci. *16*, 81–91.

Roesch, M.R., Taylor, A.R., and Schoenbaum, G. (2006). Encoding of time-discounted rewards in orbitofrontal cortex is independent of value representation. Neuron *51*, 509–520.

Ruff, C.C., Ugazio, G., and Fehr, E. (2013). Changing social norm compliance with noninvasive brain stimulation. Science *342*, 482–484.

Sapountzis, P., Schluppeck, D., Bowtell, R., and Peirce, J.W. (2010). A comparison of fMRI adaptation and multivariate pattern classification analysis in visual cortex. NeuroImage *49*, 1632–1640.

Schultz, W., Dayan, P., and Montague, P.R. (1997). A neural substrate of prediction and reward. Science *275*, 1593–1599.

Sobotka, S., and Ringo, J.L. (1994). Stimulus specific adaptation in excited but not in inhibited cells in inferotemporal cortex of macaque. Brain Res. *646*, 95–99.

Suzuki, S., Harasawa, N., Ueno, K., Gardner, J.L., Ichinohe, N., Haruno, M., Cheng, K., and Nakahara, H. (2012). Learning to simulate others' decisions. Neuron *74*, 1125–1137.

Tamir, D.I., and Mitchell, J.P. (2011). The default network distinguishes construals of proximal versus distal events. J. Cogn. Neurosci. *23*, 2945–2955.

Tanaka, S.C., Doya, K., Okada, G., Ueda, K., Okamoto, Y., and Yamawaki, S. (2004). Prediction of immediate and future rewards differentially recruits cortico-basal ganglia loops. Nat. Neurosci. *7*, 887–893.

Tomlin, D., Nedic, A., Prentice, D.A., Holmes, P., and Cohen, J.D. (2013). The neural substrates of social influence on decision making. PLoS ONE *8*, e52630.

Voon, V., Pessiglione, M., Brezing, C., Gallea, C., Fernandez, H.H., Dolan, R.J., and Hallett, M. (2010). Mechanisms underlying dopamine-mediated reward bias in compulsive behaviors. Neuron *65*, 135–142.

Wager, T.D., Davidson, M.L., Hughes, B.L., Lindquist, M.A., and Ochsner, K.N. (2008). Prefrontal-subcortical pathways mediating successful emotion regulation. Neuron *59*, 1037–1050.

Wang, Q., Luo, S., Monterosso, J., Zhang, J., Fang, X., Dong, Q., and Xue, G. (2014). Distributed value representation in the medial prefrontal cortex during intertemporal choices. J. Neurosci. *34*, 7522–7530.

Weiskopf, N., Hutton, C., Josephs, O., and Deichmann, R. (2006). Optimal EPI parameters for reduction of susceptibility-induced BOLD sensitivity losses: A whole-brain analysis at 3 T and 1.5 T. NeuroImage *33*, 493–504.

Wiggs, C.L., and Martin, A. (1998). Properties and mechanisms of perceptual priming. Curr. Opin. Neurobiol. *8*, 227–233.

Yoshida, W., Seymour, B., Friston, K.J., and Dolan, R.J. (2010). Neural mechanisms of belief inference during cooperative games. J. Neurosci. *30*, 10744–10751.

Zaki, J., Schirmer, J., and Mitchell, J.P. (2011). Social influence modulates the neural computation of value. Psychol. Sci. *22*, 894–900.

Neuron, Volume *85*

Supplemental Information

Learning-Induced Plasticity

in Medial Prefrontal Cortex

Predicts Preference Malleability

Mona M. Garvert, Michael Moutoussis, Zeb Kurth-Nelson, Timothy E.J. Behrens, and Raymond J. Dolan

Supplemental Figure S1

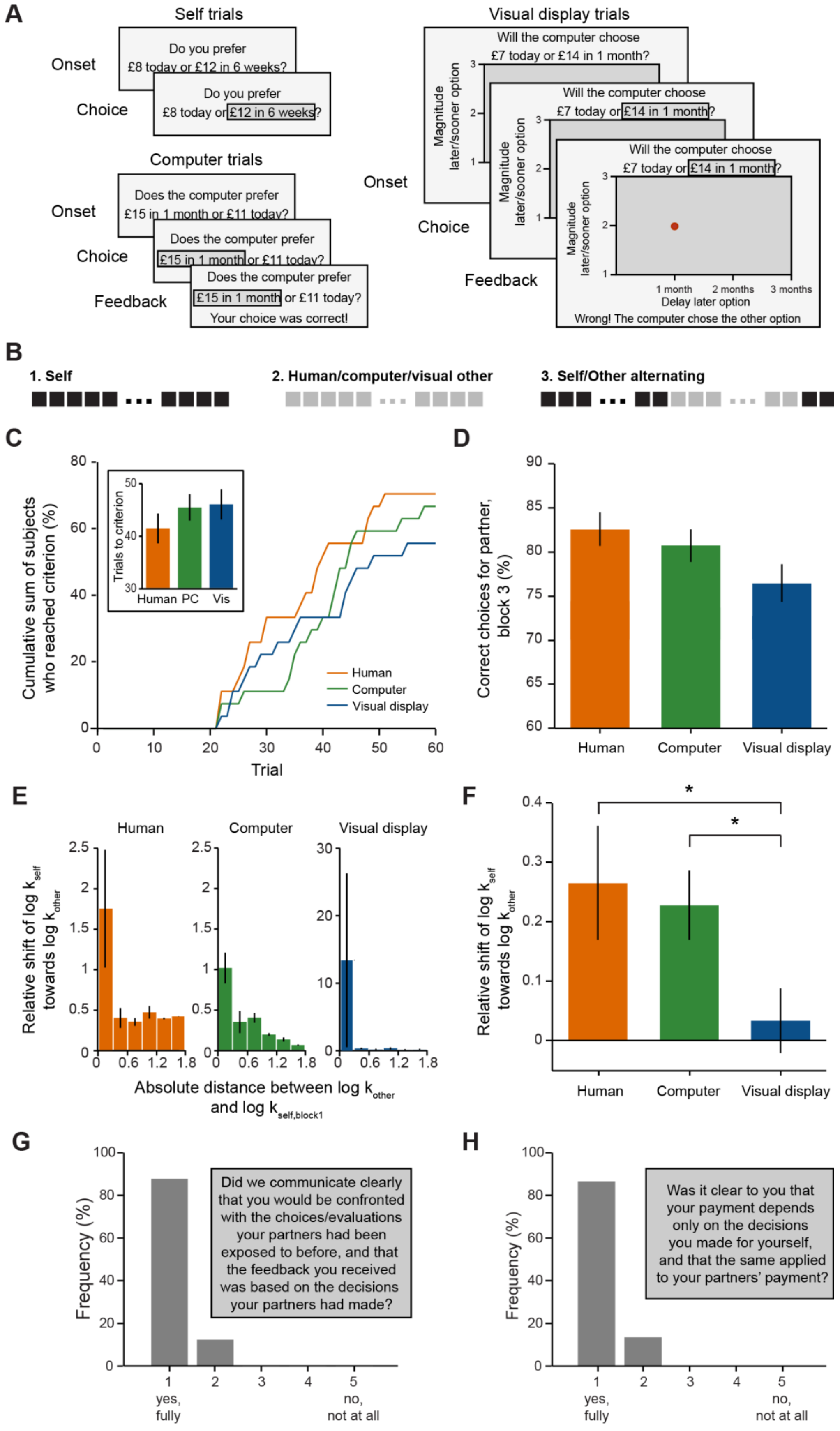

Figure S1 (related to Figure 1). Learning the preferences of another and its consequences for behaviour

(A) On self trials, subjects chose for themselves between an amount of money available on the same day and a larger amount of money available after a delay. On human and computer partner trials, subjects made these kinds of choices on behalf of their confederate. On visual display trials, the choice pair was presented on a 2D grid. Subjects were instructed to choose according to their belief about the orientation of an imaginary isoprobability line (see Supplemental Experimental Procedures for details). After each human partner, computer and visual display trial, feedback indicated whether a choice was correct.

(B) Experimental structure: Block 1 consisted of self choice trials alone, block 2 consisted of other choice trials alone and block 3 consisted of alternating short blocks of 10 choice trials per agent (self or other, see Figure 1B).

(C) Block 2 terminated after 17 correct choices for the confederate within a sliding window of 20 consecutive trials or a maximum of 60 trials. The graph depicts the cumulative sum of subjects who terminated after a given number of trials. The inset shows the mean number of trials to criterion for the different groups. The average number of trials subjects needed to reach criterion in block 2 did not differ between groups ($F_{2,78} = 0.82$, $P = 0.44$).

(D) Correct choices for the confederate in block 3 did not differ between groups ($F_{2,78} = 2.55$, $P = 0.08$). This suggests that subjects in all three groups learnt the other's discount rate equally well.

(E) Discount rate shift ($\text{shift} = \frac{\log \boldsymbol{k_{self,block\,3}} - \log \boldsymbol{k_{self,block1}}}{\log \boldsymbol{k_{other,block\,2}} - \log \boldsymbol{k_{self,block1}}}$) binned according to the distance between $|\log k_{other,block2} - \log k_{self,block1}|$. As shift estimates were inflated for $|\log k_{other,block2} - \log k_{self,block1}| \leq 0.3$, subjects who estimated the other's discount rate to be within that range were excluded from all discount rate shift analyses in the behavioural and the fMRI experiment.

(F) Shift of subjects' own discount rate in the direction of the partner's discount rate relative to the distance between $\log k_{self,block1}$ and $\log k_{other,block2}$. Subjects in the human and the computer, but not the visual display condition shifted towards the preferences of their partner ($t_{21} = 3.06$, $P = 0.006$, $t_{23} = 3.66$, $P = 0.001$ and $t_{24} = 0.61$, $P = 0.55$ respectively). A one-way ANOVA revealed that the difference in shift towards the other's preferences differed between experimental groups ($F_{2,68} = 3.5$, $P = 0.04$). Post-hoc t-tests attributed this difference to a smaller shift in the visual display group: Both subjects in the human and in the computer partner group displayed a stronger shift in discount rate towards the other than subjects in the visual display group ($t_{45} = 2.37$, $P = 0.02$ and $t_{47} = 2.25$, $P = 0.03$). There was no difference in shift towards the partner for the human versus the computer partner group ($t_{44} = 0.61$, $P = 0.5$). Subjects' estimate of the novel other's discount rate did not change from block 2 to block 3 in any of the three conditions (relative shift of other's discount rate

calculated as $\frac{\log k_{other,block\,3} - \log k_{other,block\,2}}{\log k_{self,block\,1} - \log k_{other,block\,2}}$, human partner: $t_{21} = 0.99$, $P = 0.33$, computer partner: $t_{23} = -1.75$, $P = 0.09$ and visual display: $t_{24} = -0.01$, $P = 0.99$). This confirms that discount rates for self and other are not converging. Instead the change in discount rate corresponds to a selective shift of subjects' own discount rate towards the discount rate of the other. Note we cannot rule out differences in terms of attention, working memory or other factors that prevent the update of one's own preferences for the visual display condition. Since stimuli and actions were the same as in the human and the computer partner condition, however, we can rule out that the behavioural shift in the other experimental conditions is due to simple stimulus- or action-reinforcement.

(G) After finishing the experiment, subjects completed a debriefing questionnaire designed to assess the credibility of the experimental design. Illustrated is the percentage of subjects answering 1 (yes, fully) to 5 (no, not at all) in response to the following question: Did we communicate clearly that you would be confronted with the choices and evaluations your partners had been exposed to before, and the feedback you received was based on the decisions your partners had made?

(H) Percentage of subjects answering 1 (yes, fully) to 5 (no, not at all) to the following question: Was it clear to you that your payment depends only on the decisions you made for yourself, and that the same applied to your partners' payment?

Data are represented as mean ± SEM. All post-hoc tests were Bonferroni-corrected for multiple comparisons.

Supplemental Figure S2

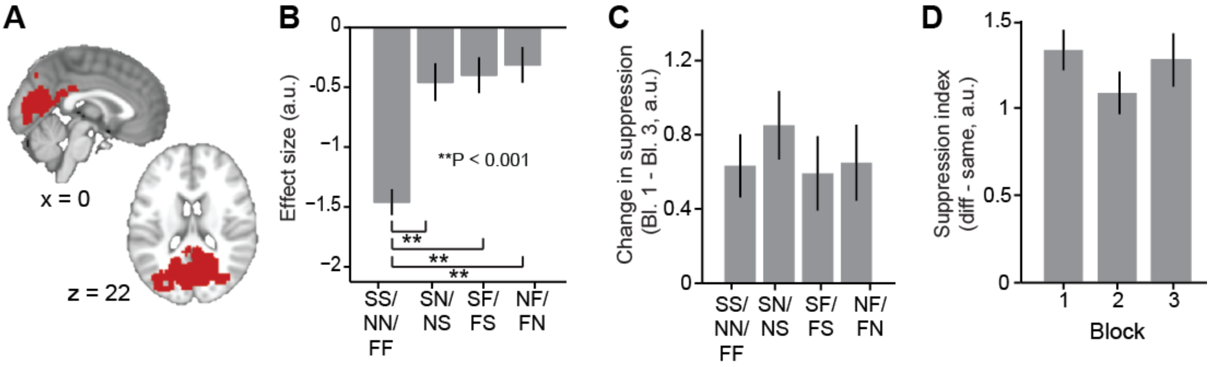

Figure S2 (related to Figure 2). Repetition suppression in visual areas

(A) Region of interest used to interrogate plasticity effects in visual regions (thresholded at $P < 0.001$ uncorrected for visualization). The region of interest was defined from a contrast indexing a main effect to any visual event in all three blocks.

(B) Visual areas displayed significantly less activity when the agent from a preceding trial was repeated than when a different agent preceded a trial ($F_{3,104} = 14.25$, $P < 0.0001$, block 1 only).

(C) Suppression increased over blocks with no difference between conditions ($F_{3,104} = 0.37$, $P = 0.78$).

(D) The difference in mean activity on same-agent-preceding trials versus different-agent-preceding trials did not change over blocks ($F_{2,78} = 0.32$, $P = 0.73$).

SS: self-preceded-by-self, NN: novel-preceded-by-novel, FF: familiar-preceded-by-familiar, SN: novel-preceded-by-self, NS: self-preceded-by-novel, SF: familiar-preceded-by-self, FS: self-preceded-by-familiar, NF: familiar-preceded-by-novel, FN: novel-preceded-by-familiar. a.u., arbitrary units.

Supplemental Figure S3

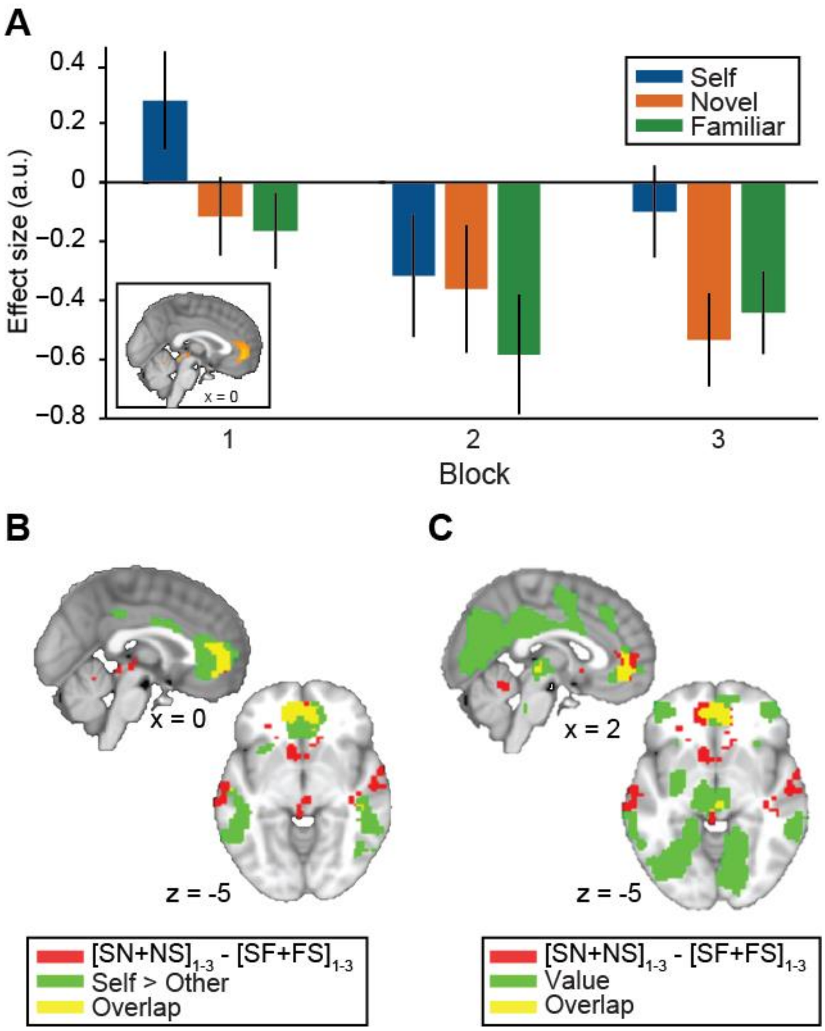

Figure S3 (related to Figure 2). MPFC activity for self, other and value

(A) Activity for self, novel and familiar other over blocks in the medial prefrontal cortex. A repeated measures ANOVA with within-subject factors “block” and “agent” showed that activity differed over blocks (F(2,52) = 13.21, P < 0.001) and between agents (F(2,52) = 4.19, P = 0.05). Furthermore, we found a block x agent interaction (F(4,104) = 3.09, P = 0.02). Post-hoc tests revealed that activity in block 1 was different from activity in blocks 2 and 3 (P = 0.005 and P < 0.001, respectively), but activity in blocks 2 and 3 did not differ (P = 0.88). Activity between familiar and novel other did not differ (P = 1.0), suggesting that the plasticity effect we report cannot be explained by differences in novelty/familiarity for the two agents. Parameter estimates were extracted from ROI shown in Figure 2B (see inset).

(B) Overlap between self > other contrast and mPFC plasticity. Mean activity on self trials was higher than on other trials in left lateral parietal cortex (P < 0.001, peak t_{26} = 6.26, peak [-39, -79, 34]) and in the mPFC (P < 0.001, peak t_{26} = 6.08, peak [9, 41, -5], thresholded at P

< 0.01 uncorrected for visualization). Activity in the mPFC overlapped with the region showing an increase in suppression between self and novel, controlled for by an increase in suppression between self and familiar as depicted in Figure 2B. The opposite contrast (other > self) only revealed activity in the visual cortex ($P < 0.001$, peak $t_{26} = 8.83$, peak [0, -94, 7], not depicted).

(C) Subjective value coding on probe trials and mPFC plasticity. Subjective value was encoded in left primary motor cortex ($P < 0.001$, peak $t_{26} = 9.54$, peak [-36, -25, 55]), in right parietal cortex ($P < 0.001$, peak $t_{26} = 5.04$, peak [54 -16 22]), in Brodmann area 10 ($P = 0.031$, peak $t_{26} = 4.37$, peak [-18, 62,7]) and in mPFC ($P = 0.055$, peak $t_{26} = 4.26$, peak [9, 44, 10], thresholded at $P < 0.01$ uncorrected for visualization). Activity in the mPFC overlapped with the region showing an increase in suppression between self and novel, controlled for by an increase in suppression between self and familiar as depicted in Figure 2B.

Results are reported at a cluster-defining threshold of $P < 0.01$ uncorrected combined with a family-wise-error (FWE) corrected significance level of $P < 0.05$. All post-hoc tests were Bonferroni-corrected for multiple comparisons. a.u., arbitrary units.

Supplemental Figure S4

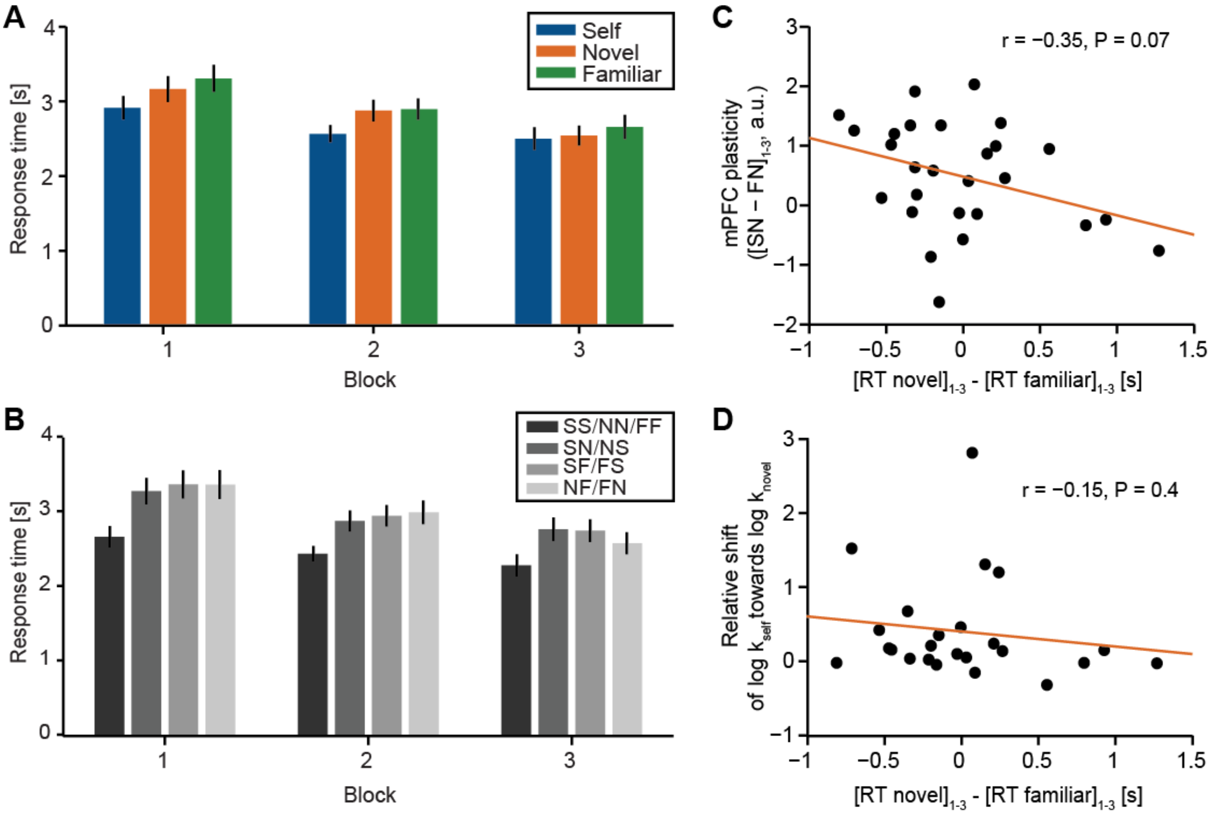

Figure S4 (related to Figure 2). Response time analyses

(A) Response times for self, novel and familiar other probe trials over blocks. A repeated measures ANOVA with within-subject factors “block” and “agent” showed different response times between blocks ($F(2,52) = 21.28$, $P < 0.001$), agents ($F(2,52) = 19.8$, $P < 0.001$) as well as a block x agent interaction ($F(4,104) = 2.88$, $P = 0.03$). Post-hoc tests reveal differences between all blocks (blocks 1/2: $P = 0.002$, blocks 1/3: $P < 0.001$, blocks 2/3: $P = 0.04$). Furthermore, subjects respond faster for self than for other ($P < 0.001$ for self/novel and self/familiar). Importantly, we found no significant difference in response times for novel and familiar other ($P = 0.29$), confirming that there is no novelty/familiarity effect.

(B) To test for behavioural suppression effects that are in line with the neural suppression effects, we performed a repeated measures ANOVA with Greenhouse-Geisser correction with within subject factors “block” and “suppression condition” (SS/NN/FF, SN/NS, SF/FS, FN/NF) on subjects’ response times on probe trials. We found a main effect of block ($F(1.442,37.500) = 21.3$, $P < 0.001$), a main effect of condition ($F(2.331,60.609) = 41.7$, $P < 0.001$), and a block x condition interaction ($F(4.571,118.859) = 4.5$, $P = 0.001$). Post-hoc paired tests revealed that SS/NN/FF differed from all other conditions ($P < 0.001$), but SN/NS, SF/FS and FN/NF did not differ from each other (all comparisons $P = 1.0$). This

emphasizes that the neural suppression effects between self and novel, and between self and familiar, respectively, cannot simply be explained by faster processing speed.

(C) Correlation between response time facilitation for the novel other (RT novel block 1 – RT novel block 3) – (RT familiar block 1 – RT familiar block 3) and $[SN\text{-}SF]_{1\text{-}3}$ plasticity effect in mPFC (ROI from Figure 2B). Response time facilitation, a crude index of increasing familiarity, shows a trend towards a negative correlation with the neural plasticity effect (R = -0.35, P = 0.07). This is in line with an observation that the opposite of familiarity, namely surprise, is a better predictor of mPFC plasticity.

(D) Response time facilitation for the novel other does not correlate with the behavioural discount rate shift of self towards the novel other (R = -0.15, P = 0.4).

All post-hoc tests were Bonferroni-corrected for multiple comparisons.

SS: self-preceded-by-self, NN: novel-preceded-by-novel, FF: familiar-preceded-by-familiar, SN: novel-preceded-by-self, NS: self-preceded-by-novel, SF: familiar-preceded-by-self, FS: self-preceded-by-familiar, NF: familiar-preceded-by-novel, FN: novel-preceded-by-familiar. a.u., arbitrary units.

Supplemental Figure S5

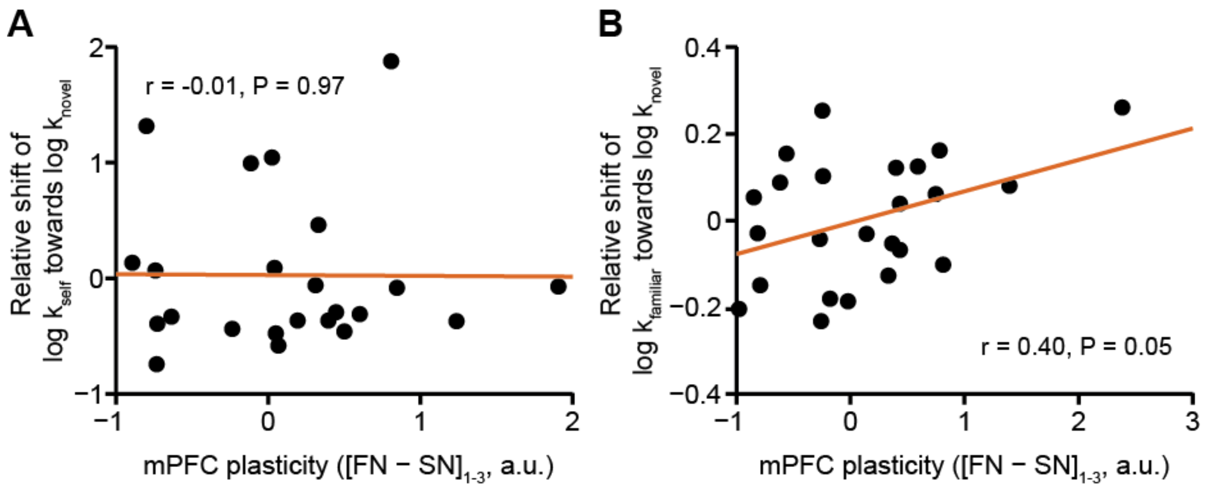

Figure S5 (related to Figure 3). Relationship between $[FN\text{-}SN]_{1\text{-}3}$ plasticity and shift in discount rate

(A) Partial correlation between the change in suppression between familiar and novel other controlled for by the change in suppression between self and novel other and the shift of subjects' own discount rate towards the novel other.

(B) Partial correlation between the change in suppression between self and novel other controlled for by the change in suppression between self and familiar other and the shift of subjects' estimate of the familiar other's discount rate towards the novel other.

Parameter estimates in A and B were extracted from the mPFC ROI shown in Figure 2B. To account for the correlation between subjects' own shift in discount rate and the shift in their estimate of the familiar other's discount rate, we performed partial correlations, i.e. the familiar shift was removed from both signals in A and the self shift was removed from both signals in B. These analyses indicate that the change of familiar-to-novel suppression versus the change in self-to-novel suppression predicted a shift in subjects' estimate of the familiar other's discount rate (partial correlation, $r = 0.40$, $P = 0.05$, Figure S5B) but not the shift in subjects' own discount rate (partial correlation, $r = -0.01$, $P = 0.97$, Figure S5A). This emphasizes the relationship between increasing neuronal similarity between two agents' value representations and increasing behavioural similarity, as depicted in Figure 3. However, note that these data are merely suggestive, as removal of the rightmost data point in Figure S5B affects the significance of the result.

a.u., arbitrary units.

Supplemental Experimental Procedures

Behavioural and fMRI task

Pairs of subjects were introduced to each other as partners before the experiment and instructed simultaneously, but performed the task in separate rooms. Both subjects made a series of choices between a smaller amount paid on the same day and a larger amount paid later (Figure S1A). The amounts varied between £1 and £20 and the delay was *tomorrow, 1 week, 2 weeks, 4 weeks, 6 weeks, 2 months,* or *3 months*. The two options were presented simultaneously and the location of the immediate and delayed option on the screen was randomized. Subjects chose by pressing a button corresponding to the location of their preferred option without any time constraint.

In block 2, subjects were instructed that they would be exposed to their partner's options from block 1 and were instructed to reproduce the partner´s decisions. Choices were correct if they corresponded to the decision that would be preferred by a hyperbolic discounter with the discount rate used to generate the decisions (see below for details).

One of the outcomes (two in the fMRI experiment) chosen by the subject for themselves was randomly selected at the end of the experiment and transferred to their bank account after the respective delay. Subjects knew that their monetary outcome depended on their own choices alone and that the choices they made for the other were not communicated to the partner and did not have any consequences for either subject (Figure S1G, H).

In the fMRI experiment, a set of probe trials was added to the experiment. The combinations of amount and delay on probe trials were drawn randomly from the same set as the options presented in choice trials. Subjects were instructed to choose according to their own preferences ("self" trials) or according to the choice their partners had made when they had participated in the same experiment previously ("other" trials).

Subjects learned the preferences of a second partner ('familiar other') before the scan (Figure 1E, top). In this pre-scan session, subjects performed one block of choices and evaluations for self before and after a block of choices for the partner. Each pre-training

block contained 48 choice trials as well as 16 randomly interleaved probe trials to familiarize subjects with this trial type. Each of the three experimental blocks in the scanner consisted of 197 probe trials for self, novel other and familiar other and 16 short interleaved blocks of one choice trial per agent (Figure 1E, bottom).

Estimation of discount rates

We estimated subjects' discount rates separately for each experimental block by fitting a hyperbolic model to their choices (Rachlin et al., 1991). On every trial, we calculated the subjective value of both options as

(1) $V = \frac{M}{1+kD}$

where V is the subjective value, M is the magnitude, D is the delay and k is a subject-specific discount rate that quantifies the devaluation of future rewards. When k = 0, subjects do not discount future rewards and base their valuation of an option purely on its magnitude. As k grows, subjects discount future rewards more and more steeply. Since the delay of the smaller option was always 0 (*today*), the subjective value of the smaller option (V_{SS}) always corresponded to its magnitude. We used a softmax function to transform the difference in subjective value between the two choice options (V_{LL}-V_{SS}) on each trial into choice probabilities:

(2) $P(LL) = \frac{1}{1+e^{-\beta(V_{LL}-V_{SS})}}$

where β is a subject-specific inverse temperature parameter that characterizes non-systematic deviations around the indifference point. We then applied maximum likelihood estimation across trials to optimize k and β. Parameter estimates were constrained such that $-4 < \log k < 0$ and $-1 < \log \beta < 1$. Note that all discount rate analyses in this paper were performed in $\log_{10}$ space, transforming typical discount rates of [0.0001 – 0] to the range [-4 – 0].

Note a similar approach also provided us with a trial-by-trial estimate of subjects' discount rates and temperature parameters. We started off with a uniform prior, which was updated

on each trial according to Bayes rule: the posterior was calculated as the likelihood of a subject’s choice given the parameters k and β weighed by the prior. This posterior was updated on subsequent trials according to the same measure. This trial-by-trial discount rate estimate could then be used to calculate the subjective value of probe trials according to Eq. 1 as well as the surprise subjects’ experienced surprise on choice trials as outlined in the Experimental Procedures. According to the same procedure, we extracted trial-by-trial estimates of the novel and familiar others’ discount rate from choices for their partners and computed the subjective value on partner trials based on these estimations (Eq. 1). This trial-by-trial value signal was used as a parametric modulator on probe trials in our fMRI GLM. Furthermore, we also estimated the surprise subjects experienced when comparing the choice they predicted a partner would make to the choice they actually observed their partners make. To this end, we applied the same procedure as outlined above, but we now used subjects’ trial-by-trial belief about the familiar and the novel other’s discount rate to estimate surprise. This measure was used as a parametric modulator in a second independent GLM which was otherwise set up exactly like the first GLM to test whether this different surprise measure would also be represented in the brain.

Optimization of choice pairs in the behavioural and scan experiment

In order to accurately estimate subjects’ shift in discount rate, we needed an efficient and precise estimate of subjects’ discount rates. To optimize choice pairs for this purpose, we alternated between two option generation methods on choice trials for self. In method 1, we first generated all possible pairs of amounts and delays and selected a subset of n/2 trials (n = total number of trials in a block) that best matched the indifference points of n/2 hypothetical subjects whose discount rates log k were evenly distributed between [-4:0] in $\log_{10}$ space (Nicolle et al., 2012). This procedure allowed for an efficient, but relatively imprecise estimate of subjects’ discount rates.

To increase the precision of our estimate of subjects’ discount rates, we alternated the thus generated trials with choices generated according to a second method, which was adaptive

in nature and based on the same Bayesian framework outlined above for estimating subjects' discount rates on a trial-by-trial basis. Here, the population distribution of log k with a mean of -2 and a standard deviation of 1 was taken as a prior belief about an individual's log k. Every time the subject made a decision, this belief distribution about their log k was updated using Bayes rule. Questions were then generated to probe our estimate of subjects' indifference point (where both options are equally preferred), which we estimated to correspond to the expected value of the current posterior. The thus generated choices were more informative about the subjects' exact discount rate than many of the other choice trials such that this procedure gave us a more precise estimate of the subjects' exact discount rate. We validated the adaptive Bayesian method against the standard non-adaptive method and found that the adaptive method produced similar results as the non-adaptive method, using fewer trials. However, data from both methods were included in the final analysis to maximize power.

Choice pairs on "other" trials, were selected as a set of trials that best matched the indifference points of 60 (block 2) vs. 100 (block 3) hypothetical subjects whose discount rates were evenly distributed across the range centred on the other's discount rate [log k_{other}-1; log k_{other}+1]. This ensured that the number of immediate and delayed choices the subject made for the partner was approximately equal.

In the fMRI experiment, choice trials were not centred on the other's discount rate, because this would have generated different kinds of choices for the novel and the familiar other. Instead, choice pairs on "other" trials in the fMRI experiment were selected as a set of trials that best matched the indifference points of 48 hypothetical subjects whose discount rates were evenly distributed across the range [-4; 0]. Choice pairs for the other were generated according to the non-adaptive method only.

Subjects in control experiment

54 volunteers (mean age ± std: 23.0 ± 7.9, 31 females) participated in the two control experiments. 27 were randomly assigned to a computer and a visual choice task condition,

respectively. There were no significant differences in age ($F_{2,78}$ = 0.71, P = 0.49) or gender ($F_{2,78}$ = 1.04, P = 0.36) between the human partner, the computer partner, and the visual display group. All subjects were neurologically and psychiatrically healthy. The study took place at the Wellcome Trust Centre for Neuroimaging in London, UK. The experimental procedure was approved by the University College London Hospitals Ethics Committee and written informed consent was obtained from all subjects.

Computer partner and visual display conditions

Subjects in the computer partner condition were told that a computer programme was trained to make decisions according to a specific strategy, while all other experimental settings were the same as in the human partner group.

Subjects assigned to the visual display condition learned a discount rate without engaging in any form of simulation. Instead, subjects were presented with a geometric depiction of a given choice on the screen (Figure S1A, right) where the x-axis of the rectangle represented the delay of the delayed option and the y-axis represented the ratio of magnitudes for the delayed and the immediate options (M_{LL}/M_{SS}). Subjects were told that the computer was programmed to choose one of the two options according to the location of the dot relative to an isoprobability line, which they had to learn based on the feedback they received after each choice. In fact, decisions in this version were generated according to the same method as for human and computer partner groups.

The orientation of the isoprobability line that subjects had to learn was determined by the discount rate k, with larger discount rates corresponding to a steeper line. This follows from comparing choices for which the value of the immediate and the delayed option are equal:

(3) $$V_{SS} = V_{LL} \Leftrightarrow M_{SS} = \frac{M_{LL}}{1+kD_{LL}} \quad \Leftrightarrow {}^{M_{LL}}/_{M_{SS}} = kD_{LL} + 1$$

Subjects were instructed that dots above the line correspond to choosing the delayed, and dots below the line correspond to choosing the immediate option. Again, choice was

translated into probabilities with a softmax function (Eq. 2) such that choices were noisy and this was communicated to the subjects.

Exclusion criterion for shift analyses

Some subjects estimated their confederate's discount rate to be very similar to their own. As a result even small shifts of subjects' own discount rate was substantially inflated when comparing it to the distance log $k_{self,block1}$ – log $k_{other,block2}$. Therefore, subjects with an absolute distance |log $k_{other,block2}$ – log $k_{self,block1}$| ≤ 0.3 were excluded from analyses. This value seemed to constitute a tipping point in all three experimental groups with strongly overrated shifts in discount rate for subjects with |log $k_{other,block2}$ – log $k_{self,block1}$| ≤ 0.3 (Figure S1F). According to this criterion, we excluded 5 subjects in the human partner condition, 3 subjects in the computer partner condition and 2 subjects in the visual display condition.

To test the robustness of our procedure to the particular threshold we chose, we examined the results for a threshold of 0.5. This excluded 12 subjects in the human partner condition, 4 subjects in the computer partner condition and 2 subjects in the visual display condition. Importantly, we still found subjects in the human and computer, but not the visual display group to shift towards the preferences of their partners: (t_{14} = 2.76, P = 0.02, t_{22} = 3.89, P = 0.001 and t_{24} = 0.61, P = 0.5, respectively) with a significant difference between groups ($F_{2,60}$ = 3.7, P = 0.03).

Scan procedure, fMRI data acquisition and pre-processing

Visual stimuli were projected onto a screen via a computer monitor. Subjects indicated their choice using an MRI-compatible button box. Stimuli were presented for a minimum duration of 3 to 5 seconds (jittered) or until subjects indicated their decision. MRI data was acquired using a 32-channel head coil on a 3 Tesla Allegra scanner (Siemens, Erlangen, Germany). A special sequence was used to acquire T2*-weighted echo-planar images (EPI) to minimize susceptibility related artefacts in the ventral prefrontal cortex (Weiskopf et al. 2006): 43 transverse slices (ascending order) of 2 mm thickness with 1-mm gap and in-plane

resolution of 3x3 mm, a repetition time of 3.01 s and an echo time of 70 ms were collected. Slices were tilted by 30° relative to the rostro-caudal axis and a local z-shim with a moment of -0.4 mT/m was applied to the OFC region. The first five volumes of each block were discarded to allow for equilibration. A T1-weighted anatomical scan with 1x1x1 mm resolution was acquired at the end of the session in order to spatially normalize the EPIs. In addition, a whole-brain fieldmap with dual echo-time images (TE1 = 10 ms, TE2 = 14.76 ms, resolution 3x3x3 mm) was obtained to correct for geometric distortions induced in the EPIs at high field strength.

We used SPM8 for image pre-processing and data analysis (Wellcome Trust Centre for Neuroimaging, London UK). We corrected for signal bias, co-registered functional scans to the first volume in the sequence and corrected for distortions using the fieldmap. Data were spatially normalized to a standard EPI template and smoothed using a 8 mm full-width at half maximum Gaussian kernel.

Physiological noise in the GLM

To reduce the contribution of physiological noise to the BOLD signal (Hutton et al. 2011), the cardiac pulse was recorded using an MRI compatible pulse oximeter (Model 8600 F0, Nonin Medical Inc., Plymouth, MN, USA) and thorax movement was monitored using a custom-made pneumatic belt positioned around the abdomen. The pneumatic pressure was converted into an analogue voltage signal using a pressure transducer (Honeywell International Inc., Morristown, NJ, USA) before digitization.

Models for cardiac and respiratory phase and their aliased harmonics were based on RETROICOR (Glover et al., 2000); the model for respiratory volume changes was based on (Birn et al., 2006). Slice 15 was used as a reference slice for modelling fluctuations arising from cardiac phase because of its proximity to the OFC (Hutton et al., 2011).

Mediation analysis

We used the used the Mediation and Moderation Toolbox (Atlas et al., 2010; Wager et al., 2008) to perform a single-level mediation analysis. To test whether the mPFC plasticity mediates the effect of striatal surprise on behavioural shift, we first extracted each individual's parameter estimate from the striatal ROI encoding surprise. The mediator corresponded to each subject's plasticity index $[SN\text{-}SF]_{1\text{-}3}$ computed from parameter estimates extracted from the mPFC ROI. The outcome variable was defined as a subject's relative shift in discount rate towards the novel other. The relationship between striatal surprise and behavioural shift controlling for the mPFC effect is referred to as path "c". We also estimated the relationship between striatal surprise and mPFC plasticity (path "a") as well as between mPFC plasticity and behavioural shift (path "b"). This last path "b" is controlled for striatal surprise, such that paths "a" and "b" correspond to two separable processes contributing to the behavioural effect. A mediation test (path "ab") examines whether the mediator (mPFC plasticity) explains a significant amount of the covariance between striatal surprise and behavioural shift. We determined two-tailed uncorrected P values from the bootstrap confidence intervals for the path coefficients (Atlas et al., 2010).

Supplemental References

Birn, R.M., Diamond, J.B., Smith, M.A., and Bandettini, P.A. (2006). Separating respiratory-variation-related fluctuations from neuronal-activity-related fluctuations in fMRI. NeuroImage *31*, 1536–1548.

Glover, G.H., Li, T.Q., and Ress, D. (2000). Image-based method for retrospective correction of physiological motion effects in fMRI: RETROICOR. Magn. Reson. Med. *44*, 162–167.
